# Can biosecurity and local network properties predict pathogen species richness in the salmonid industry?

**DOI:** 10.1371/journal.pone.0191680

**Published:** 2018-01-30

**Authors:** Tadaishi Yatabe, Simon J. More, Fiona Geoghegan, Catherine McManus, Ashley E. Hill, Beatriz Martínez-López

**Affiliations:** 1 Center for Animal Disease Modeling and Surveillance (CADMS), Department of Medicine & Epidemiology, School Veterinary Medicine, University of California, Davis, California, United States of America; 2 Centre for Veterinary Epidemiology and Risk Analysis (CVERA), UCD School of Veterinary Medicine, University College Dublin, Belfield, Dublin, Ireland; 3 Marine Institute, Rinville, Oranmore, County Galway, Ireland; 4 Marine Harvest Ireland, Rinmore, Letterkenny, County Donegal, Ireland; 5 California Animal Health and Food Safety Laboratories (CAHFS), Department of Medicine & Epidemiology, School Veterinary Medicine, University of California, Davis, California, United States of America; Aberystwyth University, UNITED KINGDOM

## Abstract

Salmonid farming in Ireland is mostly organic, which implies limited disease treatment options. This highlights the importance of biosecurity for preventing the introduction and spread of infectious agents. Similarly, the effect of local network properties on infection spread processes has rarely been evaluated. In this paper, we characterized the biosecurity of salmonid farms in Ireland using a survey, and then developed a score for benchmarking the disease risk of salmonid farms. The usefulness and validity of this score, together with farm indegree (dichotomized as ≤ 1 or > 1), were assessed through generalized Poisson regression models, in which the modeled outcome was pathogen richness, defined here as the number of different diseases affecting a farm during a year. Seawater salmon (SW salmon) farms had the highest biosecurity scores with a median (interquartile range) of 82.3 (5.4), followed by freshwater salmon (FW salmon) with 75.2 (8.2), and freshwater trout (FW trout) farms with 74.8 (4.5). For FW salmon and trout farms, the top ranked model (in terms of leave-one-out information criteria, looic) was the null model (looic = 46.1). For SW salmon farms, the best ranking model was the full model with both predictors and their interaction (looic = 33.3). Farms with a higher biosecurity score were associated with lower pathogen richness, and farms with indegree > 1 (i.e. more than one fish supplier) were associated with increased pathogen richness. The effect of the interaction between these variables was also important, showing an antagonistic effect. This would indicate that biosecurity effectiveness is achieved through a broader perspective on the subject, which includes a minimization in the number of suppliers and hence in the possibilities for infection to enter a farm. The work presented here could be used to elaborate indicators of a farm’s disease risk based on its biosecurity score and indegree, to inform risk-based disease surveillance and control strategies for private and public stakeholders.

## Introduction

**Fig 1:**
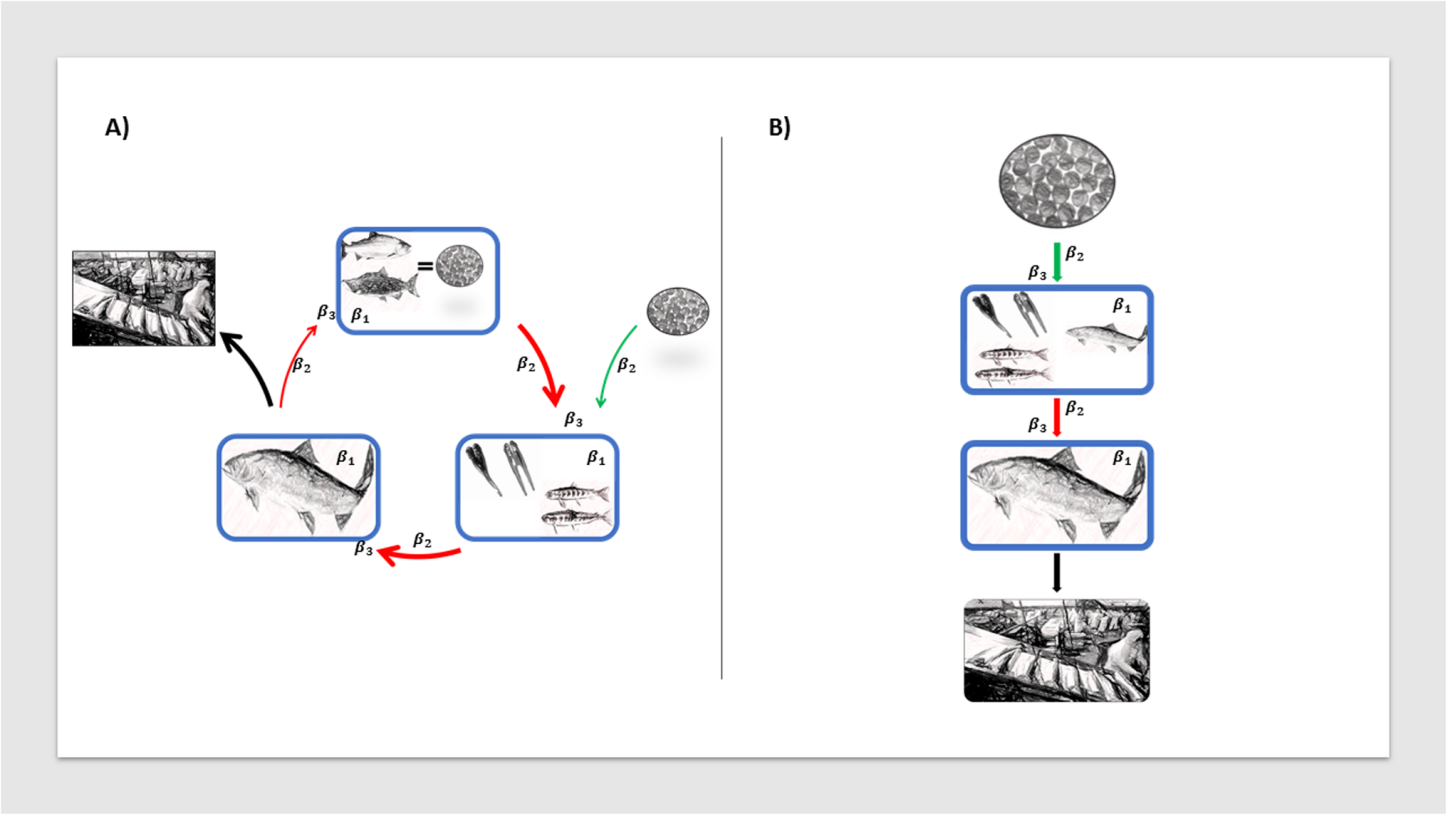
Irish salmonid production diagram. Arrows: movements of fish that remain within the system to be further grown (red), enter the system from abroad (green), or leave the system for harvest (black). Line thickness indicates quantity of fish moved. Blue boxes: protection of biosecurity against introduction and spread of pathogens within a farm, β_1_: effect of biosecurity, β_2_: effect of indegree, β_3_: effect of the interaction term between these two variables.

Commercial salmonid farming has been present in Ireland since 1979, being a significant contributor to the Irish economy, particularly along the western seaboard of the country. In Ireland, as in other countries where Atlantic salmon (*Salmo salar*) is produced, the production system is roughly divided into three types of farms: broodstock, freshwater, and seawater farms. In broodstock farms, eggs and milt are obtained from sexually mature fish to produce fertilized eggs. In freshwater farms, fertilized eggs hatch and fish are kept until smoltification, the stage where fish are ready to transition into the ocean (70–100 grams or 10–15 months of age). Some companies move the fish to net pens in freshwater lakes for the smoltification to occur there. In seawater farms, smolts are stocked and grown until they reach market size (four to five kilos at 18 to 24 months of age). Some of these fish are selected to become the broodstock for the next production cycle. In addition, some eggs and milt are imported from other European countries [1]. The rainbow trout (*Oncorhynchus mykiss*) industry in Ireland is based on egg imports from within Europe and the USA [1], which are either grown on freshwater farms until harvest, or sold to other freshwater farms for further growing, or to angler’s clubs for recreational purposes. Farms that harvest fish could either process the fish on farm or send harvested fish to be processed in farms that have a processing plant (Fig 1).

This intricate movement of fish within and between the different types of salmonid farms in Ireland could be represented as a network, and hence it is amenable for network analysis. The use of network analysis has only recently been included in the set of available tools for research of diseases of farmed salmonids, including pioneering research in Scotland [2–5], followed by our description of the network of live fish movements in Ireland [6]. However, the evaluation of local network properties as putative risk factors of salmonid diseases has not been fully explored. This is a very important area of research in aquaculture, especially considering that spread of infection via fish movement is considered one of the main routes of transmission [7, 8].

Our previous work has shown that the network of live salmonid fish movements in Ireland possesses characteristics that would facilitate infection spread processes, namely: a power-law degree distribution, short average path length and high clustering coefficients, when compared to random networks of the same order and volume [6]. This network structure determines the presence of farms that could potentially act as super-spreaders or super-receivers of disease, with few intermediaries of fish movement between farms, where infectious agents could easily spread, provided no effective barriers are placed within these farms. Additionally, all of the Irish salmon farming is certified organic, and hence farmers have a very limited therapeutic arsenal at their disposal to treat disease [9].

The above-mentioned network structure and predominant organic nature of Ireland’s salmonid farming industry highlights the importance of biosecurity, as a means of preventing the introduction and spread of infectious agents to farms within the Irish salmonid farming industry. In aquaculture, biosecurity has been defined as the sum of all procedures in place to protect living organisms from contracting, carrying, and spreading infectious agents and other non-desirable health conditions [10]. Effective biosecurity strategies provide protection to both farmed and wild aquatic animal populations, by minimizing the risk of introducing pathogens, and minimizing the consequences or further spread if the pathogen was introduced [11].

The objectives of this paper are three-fold: first, to characterize the biosecurity of the salmonid farms in Ireland, both in the freshwater and seawater environments using a survey based approach; second, based on the survey’s results, to develop a score for benchmarking the biosecurity levels of salmonid farms in Ireland; and third, to test the usefulness and validity of this score for predicting a farm’s disease risk and pathogen richness while, based on the network characteristics previously mentioned, accounting for the effect of farm centrality measures. To the authors’ knowledge, determinants of pathogen richness in an animal production setting have not previously been studied. Most of the research on this issue has been conducted in the field of ecology and emerging infectious diseases of humans [12–15]. We believe that determinants of pathogen richness could be used as variables to be included in risk-based surveillance programs [8].

## Methods

### Biosecurity characterization

For the first objective of this research, a biosecurity survey was designed, based on the Scottish Code of Good Practice [16] and the recommendations for biosecurity of the Southern Regional Aquaculture Center [17–19]. The survey was divided into the following areas: farm stocking and characteristics, predator control, cleaner fish (seawater salmon farms only), disease prevention and control, divers and diving equipment (seawater salmon farms only), handling of mortalities, feed and farm management, harvesting (seawater salmon and freshwater trout farms only), coordinated bay management and sea lice/amoebic gill disease monitoring and control (seawater salmon farms only), fish welfare and care, management of people, and biosecurity program and records. In all, there were 75 questions for Atlantic salmon freshwater farms, 108 questions for Atlantic salmon seawater farms, 89 questions for Atlantic salmon lake farms, and 80 questions for freshwater trout farms. These surveys were critically reviewed by personnel employed by industry, regulatory bodies and academia. Further, the institutional review board (IRB) administration of the University of California, Davis, determined that this research involving human subjects was exempt of an IRB review (IRB ID: 1063638–1). The surveys were piloted through administration at a research Atlantic salmon hatchery and a marine Atlantic salmon farm. Administration of the survey was conducted in person by the first author to salmonid farm managers during September and October of 2015. Informed consent was requested verbally before administering the survey. At the Atlantic salmon farms, the interview was conducted at 18 out of 20 active seawater farms, 14 out of 21 active freshwater farms, and one out of three active lake farms. At the trout farms, interviews were conducted at eight out of nine active freshwater farms. Upon arrival to freshwater salmon and trout farms, a walkthrough of the premises was carried out, which lasted between 15 to 30 minutes. For salmon seawater farms, the interviewer was transported via boat to inspect the net pens and meet with the manager. The duration of the survey administration ranged between one and two hours. The surveys are available upon request to the corresponding author.

### Biosecurity scoring system

For the second objective of this paper, a score for each farm was calculated based on the manager’s response to each of the questionnaire’s close-ended questions. Each question had a maximum attainable score (i.e. the response(s) that was deemed, based on the above mentioned references, to make a farm least vulnerable to disease introduction and spread), from which points were discounted if other, less optimal options were being carried out at the farm. For example, for the question “Does the farm receive fish from other seawater farms on a typical production cycle?” the maximum attainable score was one point if the answer to this question was ‘no’. Another example for seawater farms is the question “How do you make sure fish that arrive at your farm are in good condition?” A farm would get a maximum score of six points for this question if each of the following were requested by the farm manager: health certificates, diagnostic test results, a sanitary history, and smoltification test results from its suppliers, together with inspection prior to purchase and upon arrival. One point would be deducted from this maximum score for each of these measures that were not reported by the farm manager.

Each question had two quantities associated with it: the score obtained by the farm, and the maximum potential score attainable. Therefore, the computation of the overall biosecurity score for a farm *i* was based on the following formula

$$\frac{\sum obtained\;points_{i}}{\sum potential\;points_{i}}\times 100=biosecurity\;score_{i}$$ (1)

Where the score is scaled by 100 to make it a percentage of the “maximum attainable biosecurity”. Open-ended questions were excluded as they were few in number, mostly descriptive, and with varying levels of completion or detail by interviewed managers, and hence not readily amenable to a scoring system of this kind. The above described score makes the simplifying assumption that all the included items have the same impact on biosecurity.

### Pathogen richness models

The only open-ended question from the survey that was kept, although not used in the scoring of a farm, was the list of different diseases that occurred in the farm in the 12 months prior to the survey (subsequently termed pathogen richness). This refers to manifest signs of disease, which include overt fish mortality and the attributed disease causing agent. These diagnoses were mainly made by a fish health specialist. Pathogen richness was modeled as originating from a generalized (or Lagrangian) Poisson process, as defined by Consul and Jain [20], with a probability mass function given by

$$P\left(N=n\right)=p_{n}\left(\theta ,\lambda ,m\right)=\{\begin{array}{c} \theta {\left(\theta +n\lambda \right)}^{n-1\frac{\text{exp}\left(-\theta -n\lambda \right)}{n}}\\ 0 \end{array}\begin{array}{l} for\;n=0,\;1,\;2,\ldots ,\;m\\ for\;n>m\;when\;\lambda <0 \end{array}$$ (2)

Where *θ* > 0, max(-1,-θm)≤λ≤1, and *m* taken equal to the largest possible integer such that *θ* + *mλ* > 0 when *λ* is negative [21]. The expectation and variance of this distribution are given by

$$E\left(N\right)=\frac{\theta }{1-\lambda },\;Var\left(N\right)=\frac{\theta }{{\left(1-\lambda \right)}^{3}}$$ (3)

From this, it can be seen that this distribution allows data to be modeled that shows either over-dispersion (for which *λ* > 0) or under-dispersion (for which *λ* < 0), and that the generalized Poisson distribution (GPD) reduces to the Poisson distribution when *λ* = 0 [22].

From an interpretative point of view, this distribution extends the Poisson distribution by its ability to describe situations where the probability of occurrence of a single event does not remain constant (as in a Poisson process), but is affected by previous occurrences [23]. The generalized Poisson distribution has been found to accurately describe phenomena as diverse as the observed number of industrial accidents and injuries, where a learning effect may be present; and the spatial distribution of insects, where initial occupation of a spot by a member of the species has an influence on the attractiveness of that spot to other members of the species [24]. Similarly, the number of different diseases affecting a farm could be thought of as arising in a similar manner, where the occurrence of an outbreak of disease is influenced by other disease events that have previously occurred on the farm. This effect could arise from synergy or antagonism between agents, or from a learning curve or changes in the level of awareness in farm personnel.

To assess the effect of in-farm biosecurity (measured as a biosecurity score) and farm centrality (specifically indegree) on pathogen richness, a generalized Poisson regression model [22] was fit to the data of the form

$$\text{log}\left(E\left(N|biosec,\;indegree\right)\right)=\text{log}\left(\frac{\theta }{1-\lambda }\right)=\beta_{0}+\beta_{1}biosecurity+\beta_{2}indegree+\beta_{3}interaction$$ (4)

Where *E*(*N*|*biosec*, *indegree*) is the expected pathogen richness given the farm biosecurity score and indegree, *θ* = exp(*β*_0_ + *β*_1_ *biosecurity* + *β*_2_ *indegree* + *β*_3_ *interaction*)(1 − *λ*), *β*_0_ is the intercept, *β*_1_ is the regression coefficient for the farm’s biosecurity score, *β*_2_ is the regression coefficient for the farm’s indegree, and *β*_3_ is the regression coefficient for the interaction between biosecurity and indegree (Fig 1 and Table 1). The values of *λ* and *θ* were restricted as described above in the support of the distribution.

**Table 1:** Variables evaluated in the pathogen richness models.

Variable name	Definition	Theoretical range
Biosecurity score	Score obtained from assigning points to items of the biosecurity survey applied to farm managers	0 to 100
Indegree	Number of different farms within the country from which a farm receives fish	0 to n-1
Pathogen richness	Number of different pathogens attributed to disease outbreaks in preceding year	0 to the number of pathogens present in the country for the specific environment (FW/SW) and host species

n: number of farms (salmon or trout) in the network; FW: freshwater; SW: seawater

Separate models were created for seawater salmon farm (SW) and for freshwater farms (FW), with the latter including salmon hatcheries, lake farms, and freshwater trout farms. The largest possible value, m, was set equal to three as this was the maximum number of diseases reported in these types of farms during the survey.

Model fitting was carried out in a Bayesian framework, with priors

$$\beta_{0},\;\beta_{1},\beta_{2},\beta_{3}\sim \;Normal\left(0,\;0.5\right)$$ (5)

λ~Normal(0,1) (6)

Indegree was transformed into a binary variable, with a value of 0 for a farm with indegree = 1 (i.e. only one fish supplier), and 1 for a farm with indegree > 1 (i.e. two or more fish suppliers) during the year prior to the survey (2014). For three farms that did not report any fish movements during 2014, data from 2013 was used. The biosecurity score was scaled to have a mean of 0 and standard deviation of 0.5, and dichotomized indegree was centered at its mean. These transformations allow for the interpretation of the regression coefficients to be more transparent, by making them directly comparable from a parameter estimates table [25], although plotting is a much clearer means of understanding the effects and interactions of the predictor variables, and hence was the main approach used here. Separately for SW and FW, five models were fit: i) the full model with two main effects (biosecurity score and indegree) and their interaction, ii) a main effects (no interaction) model, iii) a model with biosecurity alone, iv) a model with indegree alone, and v) a null model with no predictors in it. Comparisons between these models were done using the leave-one-out cross-validation information criterion (looic) [26].

Each model was initially fitted using four chains of 4,000 iterations with a warm-up of 2,000 iterations for assessing model convergence, after which an individual chain of 16,000 iterations with warm-up of 8,000 iterations was used for inference for each model. Model convergence diagnostics included visual checking of trace plots, to visually evaluate stationarity and mixing of the chains, Gelman-Rubin convergence diagnostic, $\hat{R}$, and the number of effective samples [27, 28].

### Binary logistic regression models

For each disease composing the pathogen richness index of SW and FW farms, separate binary logistic regression models were fit with the same priors for the intercepts and regression coefficients which, in addition to the above mentioned variables, included the other diseases as covariates, to further explore the results of the pathogen richness models.

The models were fit using Stan’s Hamiltonian Monte Carlo sampling [29] in the R statistical environment [30], using the Rstan package [31]. Leave-one-out cross-validations were done using the loo package in R [32]. Posterior predictive checks were used to evaluate if simulated samples from the best fitting pathogen richness models (based on looic) matched the original data. This was done visually through hanging and suspended rootograms [33], and comparing the mean and variance of simulated and observed data. For generating random samples from a generalized Poisson distribution, the package RMKdiscrete [34] was used.

## Results

### Biosecurity characterization

In total, 21 farm managers completed the survey. This corresponds to 35 farms (66% of active salmonid farms in Ireland during the survey period), as some managers were in charge of two or more farms. Of these farms, 27 (77%) were Atlantic salmon and eight (23%) were freshwater trout farms. Of the salmon farms, 18 were seawater farms, eight were freshwater farms, and one was a freshwater lake farm. Most of the trout farms were inland farms where fish were produced for grow out in other inland farms or for consumption, except for a small scale farm which raised trout for repopulation purposes. One of the seawater salmon farms was fallowed since 2013, so it was not further considered for the pathogen richness models, which were then based on data from 17 farms. Biosecurity survey results are included in supporting information S1, S2 and S3 Files for Atlantic salmon seawater, Atlantic salmon freshwater, and freshwater trout farms, respectively.

### Biosecurity score, indegree, and pathogen richness

Regarding the biosecurity score, in general seawater salmon farms had the largest values, followed by freshwater salmon farms and trout farms, the latter two being very similar. Indegree was also highest among seawater farms, followed by freshwater salmon farms, with freshwater trout farms being the lowest. With respect to pathogen richness, the largest values were for freshwater trout and seawater salmon farms, with freshwater salmon farms showing the least richness (Table 2).

**Table 2:** Descriptive statistics for the biosecurity score, indegree and pathogen richness for the surveyed farms.

Type (N)	Biosecurity score			Indegree			Pathogen richness		
	1^st^ quartile	Median	3^rd^ quartile	1^st^ quartile	Median	3^rd^ quartile	1^st^ quartile	Median	3^rd^ quartile
SW salmon (18*)	82.3	84.1	87.7	1.0	2.0	2.0	1.0	1.0	2.0
FW salmon (8)	70.3	75.2	78.5	0.8	1.0	2.0	0.0	0.0	0.3
FW lake salmon (1)	NA	94.4	NA	NA	1.0	NA	NA	0.0	NA
FW trout (8)	71.8	74.8	76.3	0.0	0.5	1.0	1.0	1.0	2.0

SW: seawater; FW: freshwater;

* indegree and pathogen richness scores based only on 17 seawater salmon farms

The most common disease affecting seawater salmon farms was pancreas disease (PD) caused by the salmonid alphavirus (SAV; 14 farms), followed by amoebic gill disease (AGD) caused by *Neoparamoeba perurans* (10 farms), with one farm reporting the occurrence of an infectious pancreatic necrosis (IPN) outbreak. Only one farm reported that it had not experienced any disease outbreak in the preceding year, eight farms reported experiencing one disease (either PD or AGD), seven farms reported experiencing two diseases (both PD and AGD), and one farm reported experiencing all three diseases in the previous year. For freshwater salmon farms, only two farms reported experiencing disease(s) in the preceding year, one farm reported an outbreak of furunculosis (*Aeromona salmonicida subsp*. *salmonicida*), and the other one reported three diseases: *Ichthyobodo sp*., and unspecified gill and fungal infections. Except for one farm, all freshwater trout farms reported experiencing disease in the preceding year: two farms reported bacterial gill disease (BGD) caused by *Flavobacterium branchiophilum*, one farm reported rainbow trout fry syndrome (RTFS) caused by *Flavobacterium psychrophilum*, and four farms reported both RTFS and *Ichthyobodo sp* (Table 3).

**Table 3:** Reported pathogens causing disease among Irish salmonid farms and distribution of pathogen richness during Sep 2013 –Oct 2014, by type of farm.

Type (N)	Disease causing agents (N)	Pathogen richness of 0, 1, 2, and 3 (N)
SW salmon (17*)	SAV (14), *N*. *perurans* (10), IPNv (1)	0 (1), 1(8), 2(7), 3(1)
FW salmon (8)	*A*. *salmonicida subsp*. *Salmonicida* (1), *Ichthyobodo sp*. (1), unspecified gill (1) and fungal (1) infections	0 (6), 1 (1), 3 (1)
FW lake salmon (1)	None	0 (1)
FW trout (8)	*Flavobacterium branchiophilum* (2), *Flavobacterium psychrophilum* (5), *Ichthyobodo sp* (4)	0 (1), 1 (3), 2 (4)

SW: seawater; FW: freshwater;

* disease outbreaks reported only on 17 seawater farms; SAV: salmonid alphavirus; IPNv: infectious pancreatic necrosis virus

### Pathogen richness models

For the pathogen richness models, model comparisons are presented in Table 4 for the two settings (seawater and freshwater). For the seawater farms, the best ranking model was the full model (looic = 33.3), which included the biosecurity score, indegree, and their interaction.

**Table 4:** Model comparisons for pathogen richness models for seawater and freshwater farms using leave-one-out information criterion (looic).

**Seawater farms (N = 17)**				
**Model**	**looic**	**SE looic**	**p-loo**	**SE p-loo**
Full model	33.3	3.1	1.9	0.7
Biosecurity score	39.3	4.1	1.8	0.8
Null model	39.9	5.3	1.5	0.7
Main effects	40.1	4.2	2.3	0.8
Indegree	41.4	5.4	2.1	0.9
**Freshwater farms (N = 17)**				
Null model	46.1	5.4	1.5	0.3
Biosecurity score	46.2	5.3	1.7	0.3
Indegree	46.8	5.4	1.8	0.3
Main effect	47.0	5.3	2.0	0.3
Full model	47.3	5.4	2.2	0.3

p-loo: number of effective parameters

**Fig 2:**
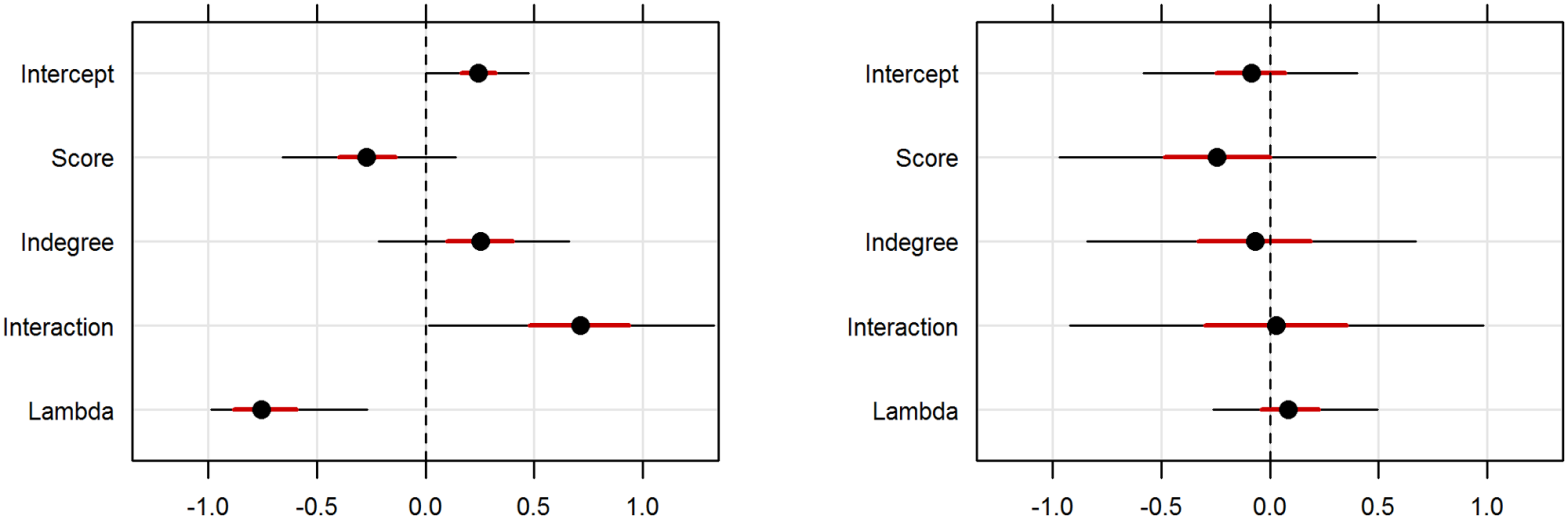
Probability distribution of the parameters of the top ranked seawater farm model (left), and equivalent model for freshwater farms (right). Black dot: median, red thick line: 50% PI, black thin line: 95% PI.

**Fig 3:**
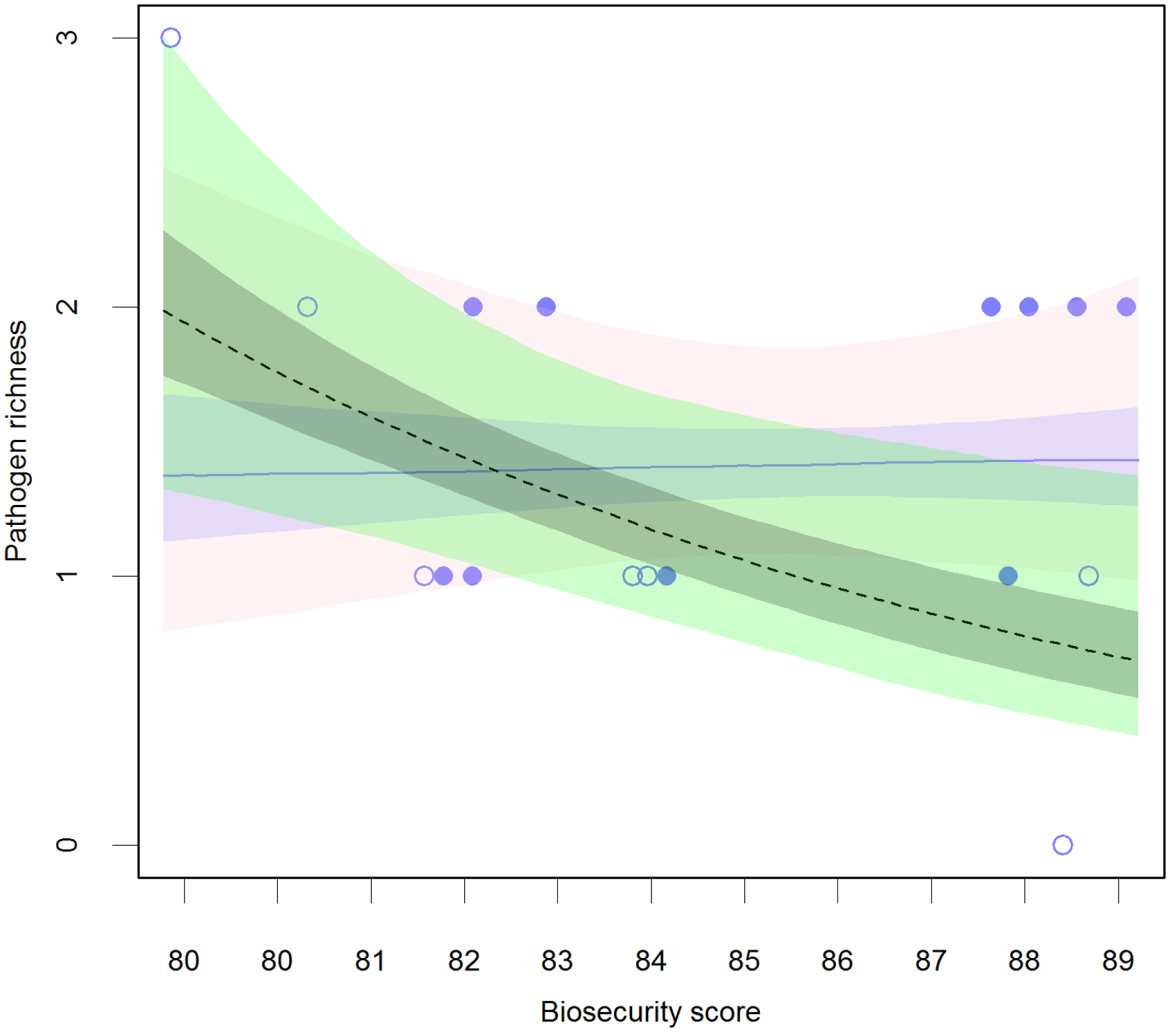
Model’s estimate of the mean pathogen richness affecting a seawater farm during a year for farms with high (i.e. > 1) and low (i.e. = 1) indegree. Farm with a high indegree: median (solid blue line), 50% PI (purple shaded area), and 95% PI (pink shaded area); Farm with a low indegree: median (dashed grey line), 50% PI (grey shaded area), and 95% PI (green shaded area). Observed data for farms with a high indegree (filled points) and low indegree (hollow points). A jitter was added to the observed data points to avoid superimposition on the plot.

For the latter model, both the biosecurity score and indegree seemed to be important predictors (most of the probability mass was away from the null value of zero), with farms having a higher biosecurity score being associated with a lower pathogen richness affecting the farm, and farms with indegree > 1 being associated with an increased pathogen richness. The effect of the interaction between both variables also seemed to be important, indicating that these two variables modulate each other’s effect (Table 5 and Figs 2 and 3).

**Table 5:** Parameter estimate’s posterior distribution of pathogen richness’ top ranked seawater model and an equivalent model for freshwater farms.

**Seawater farms (N = 17)**						
**Parameter**	**mean**	**SD**	**2.5%**	**25%**	**75%**	**97.5%**
Intercept	0.24	0.12	0.01	0.16	0.32	0.47
Biosecurity score	-0.27	0.20	-0.66	-0.40	-0.14	0.14
Indegree	0.25	0.22	-0.22	0.10	0.40	0.66
Interaction	0.70	0.34	0.02	0.48	0.94	1.33
lambda	-0.72	0.20	-0.99	-0.88	-0.59	-0.27
**Freshwater farms (N = 17)**						
Intercept	-0.09	0.25	-0.58	-0.25	0.07	0.40
Biosecurity score	-0.24	0.36	-0.97	-0.49	0.00	0.49
Indegree	-0.07	0.39	-0.84	-0.33	0.19	0.67
Interaction	0.03	0.48	-0.92	-0.30	0.36	0.99
lambda	0.10	0.20	-0.26	-0.04	0.23	0.50

For the effect of biosecurity, although the 95% PI crosses the null value of zero (95% PI: -0.66, 0.14), most of the posterior distribution lies below this value (50% PI: -0.40, -0.14), with a mean of -0.27. Similarly, the posterior distribution of the effect of indegree is mostly above zero (95% PI -0.22, 0.66; 50% PI: 0.10, 0.40), with a mean of 0.25. In the case of the interaction term between these two variables, it has a median of 0.70 (95% PI: 0.02, 1.33; 50% PI: 0.48, 0.94). Lambda, the dispersion parameter of the distribution had a mean posterior value of -0.72 (95% PI -0.99, -0.27; 50% PI: -0.88, -0.59), indicating that the pathogen richness experienced by a seawater farm could be considered an under-dispersed Poisson process. For the freshwater farms, the best ranking model was the null model (leave-one-out cross-validation information criteria, looic = 46.1), indicating that none of the predictors might be associated with the number of diseases affecting a freshwater farm, although there was negligible difference with the model with only biosecurity score (looic = 46.2). The estimated effect of biosecurity for the full FW model is very similar to the one estimated for SW model, mean of -0.24, although the uncertainty is much higher (95% PI: -0.97, 0.49; 50% PI: -0.49, 0.00) (Table 5, Fig 2). Here lambda had a mean posterior of 0.10 (95% PI -0.26, 0.50; 50% PI: -0.04, 0.23), indicating that the pathogen richness experienced by a freshwater farm could be thought of as an over-dispersed Poisson process.

These results are in support of the hypothesis that pathogen richness in SW farms is affected by biosecurity, indegree and their interaction. Further, that pathogen richness in FW farms is likely affected by biosecurity. The effects of these variables in SW farms are further explored in Fig 3. This figure shows that the effect of biosecurity is modulated by the farm’s indegree. If indegree is low (i.e. only one fish supplier), biosecurity seems to have a protective effect, reducing the expected pathogen richness affecting a seawater farm, as shown by the black dashed line on the plot. On the other hand if indegree is high (i.e. more than one supplier of fish), the effect of an increasing biosecurity seems to be negligible, as shown by the mostly flat blue horizontal line.

**Fig 4:**
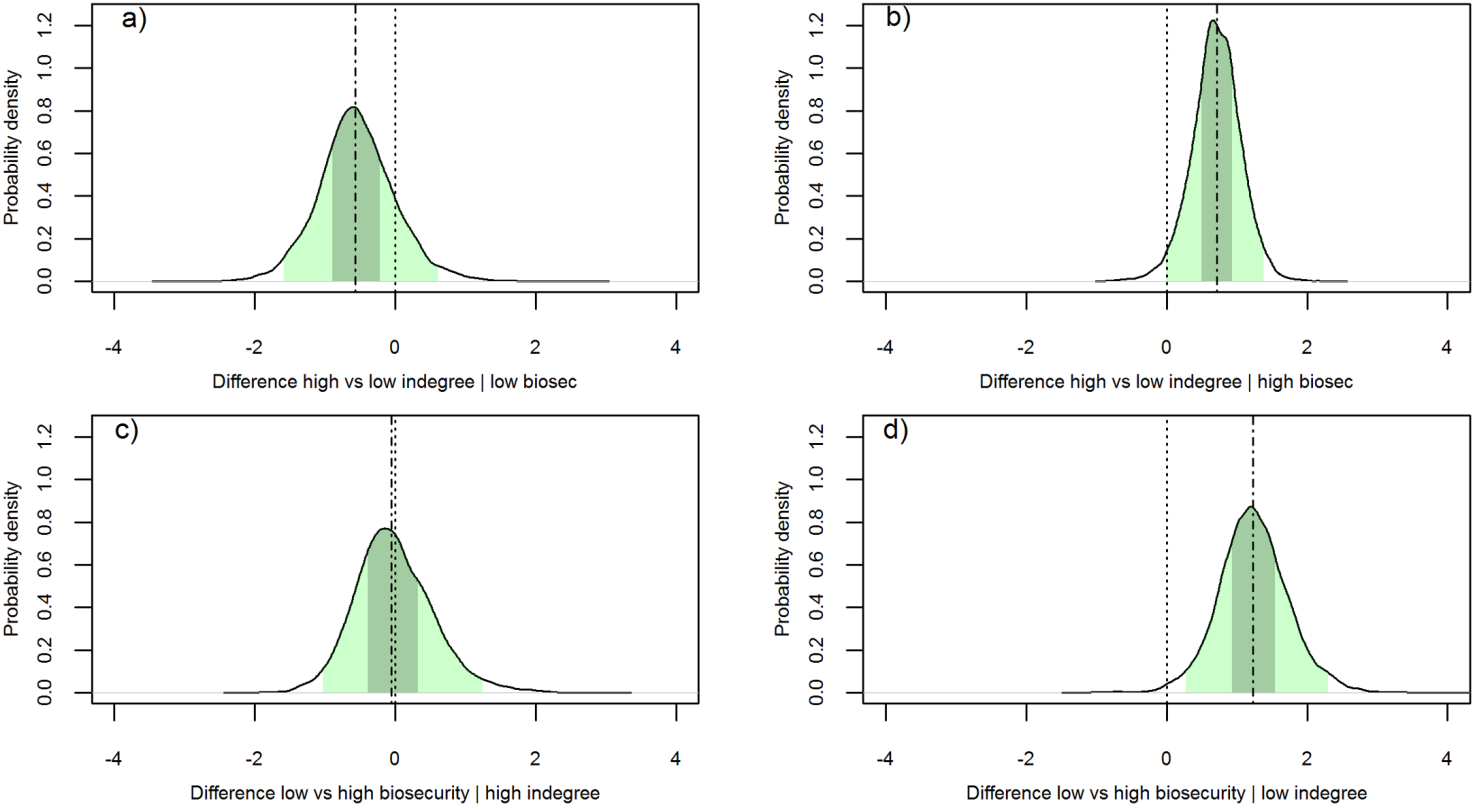
Posterior density of the difference in mean pathogen richness of a seawater salmon farm during a year. a) high (i.e. > 1) vs low indegree (i.e. = 1) given a low (79.5) biosecurity score, b) high vs low indegree given a high (88.6) biosecurity score, c) low vs high biosecurity given a high indegree, d) low vs high biosecurity given a low indegree. Green shaded area: 95% PI of the difference; grey shaded area: 50% PI of the difference; dashed vertical line: median difference; dotted vertical line: a null difference of 0.

Fig 4 further explores this interaction, showing the estimated difference in mean pathogen richness of farms when one of the variables (biosecurity score or indegree) varies while keeping the other constant. Specifically, the upper left plot shows the difference for a farm with high indegree versus a farm with a low indegree, when both farms have a low biosecurity score: here we see a median difference of -0.57 (95% PI: -1.60, 0.61; 50% PI: -0.89, -0.22). On the other hand, the upper right plot shows the same comparison for farms with high biosecurity, with a median difference in the rate of 0.71 (95% PI: -0.01, 1.38; 50% PI: 0.49, 0.93). Regarding the effect of differences in biosecurity, the lower left plot shows that for farms with high indegree the difference between farms with low and high biosecurity is almost inexistent, with median of -0.06 (95% PI: -1.03, 1.24, 50% PI: -0.39, 0.32), while for farms with low indegree (lower right plot), the difference is substantial, with a median of 1.22 (95% PI: 0.26, 2.30; 50% PI: 0.92, 1.54).

**Fig 5:**
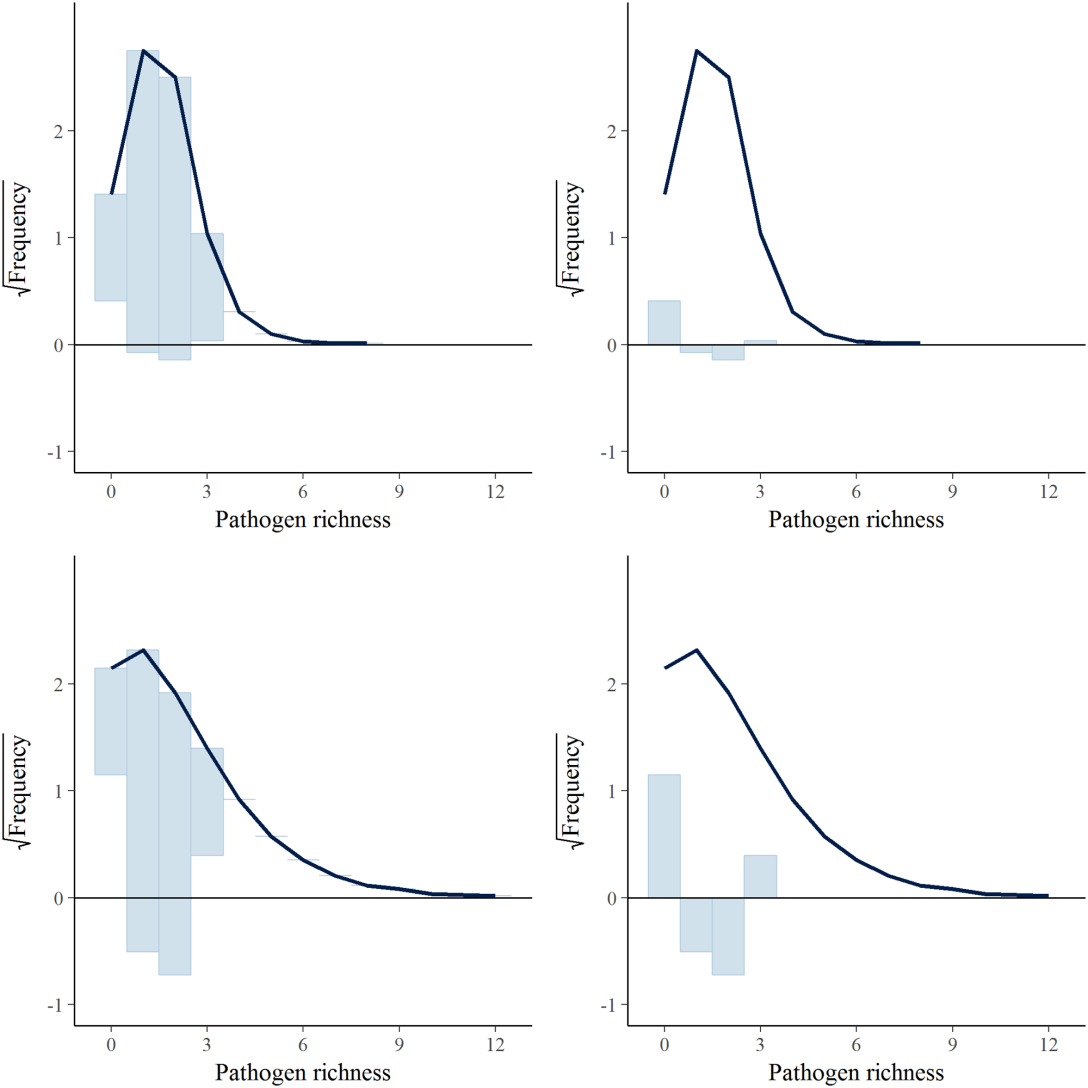
Rootogram of observed and simulated pathogen richness affecting a seawater salmon farm during a year. Top: hanging (left) and suspended (right) rootograms for the full generalized Poisson regression model. Bottom: hanging (left) and suspended (right) rootograms for an equivalent Poisson regression model.

Fig 5 shows hanging (left) and suspended (right) rootograms comparing the distribution of observed (bars) and simulated (thick line) values of pathogen richness affecting the surveyed seawater salmon farms during a year. Briefly, in a hanging rootogram, frequency bars of observed counts are “hanging” from the curve representing the mean expected counts. The gap between the lower end of the bars and the horizontal zero line represents the discrepancies between observed and expected frequencies. In a suspended rootogram, these differences are represented as bars of a histogram [33, 35]. In Fig 5, the top row shows the simulated counts from the generalized Poisson model fitted to seawater farms, while the bottom row shows the simulations from an equivalent Poisson regression model, where mean and variance are assumed to be equal. In the former, it can be seen that the model overestimates the zero counts, with a mean difference in the proportion of simulated versus observed counts of 5.8%, with slightly biased estimates for the proportion of counts equal to one, two, and three with mean differences of -2.5%, 4.3%, and 0.4%, respectively. On average 0.6% of the simulated counts from this model were greater than the maximum observed value of three, with a maximum simulated count of eight. In the case of the Poisson regression model, the proportion of zero counts is greatly overestimated with a mean difference in the proportion of simulated versus observed counts of 21.3%, also producing more biased simulated counts of one, two, and three with mean differences of -15.4%, -19.5%, and 5.6%, respectively. On average 8.0% of simulated counts from this model were greater than the maximum value of three, with a maximum simulated count of 12. For the generalized Poisson model, the simulated counts had a mean of 1.40 (95% PI: 0.94, 1.88; 50% PI: 1.24, 1.53) and variance of 0.63 (95% PI: 0.25, 1.32; 50% PI: 0.44, 0.76). These results are in line with the observed data, which had a mean of 1.47 and a variance of 0.51. On the other hand, for the Poisson regression model the mean and variance were 1.46 (95% PI: 0.76, 2.30; 50% PI: 1.17, 1.71) and 1.78 (95% PI: 0.57, 4.10; 50% PI: 1.13, 2.22), respectively.

### Binary logistic regression models

For the diseases occurring in seawater, Binary logistic regression models were evaluated only for PD and AGD. IPN was excluded from this analysis, as it had only one case reported. Both of these models had all of their regression coefficients with 95% PIs including the null value of zero. Hence, we will refer to the most notable findings based on a 50% PI. Detailed model output can be found in supporting information S4. For the PD model, the biosecurity score was the only covariate associated with outbreaks of this disease at the 50% PI level, with a mean posterior of -0.36 (95% PI: -1.24, 0.53; 50% PI: -0.67, -0.04). The occurrence of AGD outbreaks was borderline associated with PD, with a mean posterior of 0.26 (95% PI: -0.59, 1.09; 50% PI: -0.04, 0.55). There were no evident associations at the 50% PI level for the AGD model for the evaluated covariates or with the occurrence of PD outbreaks.

For the diseases occurring in freshwater, binary logistic regression models were evaluated only for BGD, *Ichthyobodo* sp., and RTFS, excluding furunculosis and fungal infection, as there were only single occurrences reported of these diseases. As for the SW diseases, the 95% PI of all regression coefficients crossed the null value of zero. As previously, we will refer here to the most notable findings at the 50% PI level, with detailed model output available in supporting information S4. For the BGD model, there were two covariates that seemed possibly associated with the occurrence of outbreaks of this disease: the biosecurity score, with a mean posterior of -0.35 (95% PI: -1.24, 0.54; 50% PI: -0.66, -0.05), and the occurrence of RTFS outbreaks, with a mean posterior of -0.34 (95% PI: -1.25, 0.57; 50% PI: -0.66, -0.03). For the *Ichthyobodo* sp. outbreak model, the only covariate that seemed possibly associated with the occurrence of outbreaks of this disease was the occurrence of RTFS, with a mean posterior of 0.40 (95% PI: -0.48, 1.28; 50% PI: 0.10, 0.71). Similarly, for the RTFS model the only covariate that seemed possibly associated with the occurrence of outbreaks of this disease at the 50% PI level was the occurrence of *Ichthyobodo* sp. outbreaks, with a mean posterior of 0.41 (95% PI: -0.46–1.29; 50% PI: 0.11–0.71).

## Discussion

There are few published examples of biosecurity evaluation in the finfish farming industry, the work of Delabbio *et al*. [36–38] being a notable exception. These authors first characterized the biosecurity of the recirculation sector of finfish aquaculture in the US and Canada, and then carried out an assessment of the attitudes, beliefs, and perceptions of managers and owners towards fish diseases and biosecurity. They found that biosecurity utilization in these countries is neither consistent nor uniform, and that perceptions about risk varied widely, with the majority of respondents thinking that infections coming in with new fish were the most serious introduction risk to their farm. Our results are similar regarding the heterogeneity with which biosecurity is applied (Table 2), adding a quantitative association between biosecurity, incoming new fish (indegree) and disease risk for seawater farms (Table 5, Figs 2, 3 and 4). Nevertheless, exploration of the human dimension of the application of biosecurity practices at the farm level is still lacking in Ireland. Acknowledgment of the human dimensions aspect of biosecurity utilization is important in the creation of biosecurity programs, strategies, and policies that will be accepted, implemented, and consistently applied by commercial fish farmers [38].

In other livestock industries, this type of research has found that farmers associate farm-level biosecurity with positive outcomes including improved profitability, professional pride and good reputation. Concurrently, farmers question the efficacy of these measures in the absence of action by others, and they consider the burden of these measures to be excessive on them. On the side of veterinary surgeons, there also seems to be major concerns regarding the efficacy of biosecurity [39]. Our results support the view of biosecurity as an important measure to decrease pathogen incidence, either in the form of specific diseases (PD and BGD in the marine and freshwater environments respectively, supporting information S4) or as pathogen richness in both marine and freshwater environments (Table 5). Further, these results suggest that the use of codes of good practice for improving farm biosecurity, like the one from Scotland, would be beneficial in reducing pathogen richness and disease incidence faced by fish farmers.

Qualitative analysis has also identified important attitudes and beliefs as influencers of behavior regarding control of highly contagious animal diseases, in addition to other factors such as trust in neighbors and regulatory agencies, moral norms, and risk perception[40]. It is the impression of the authors that these constraints are also an important determinant of the heterogeneity in the application of biosecurity in the Irish salmonid farming industry. This warrants further research in this area.

Regarding the effect of indegree, our results demonstrate that seawater farms with low indegree would have lower pathogen richness during the production cycle (Table 5, Fig 2). Nevertheless, the protective effect of a low indegree would only be manifest for marine farms with high biosecurity levels (Fig 4a and 4b). Similarly, the protective effect of high biosecurity would only be present for farms with low indegree (no more than one fish provider) (Fig 4c and 4d). This antagonistic effect between biosecurity and indegree seems very meaningful, suggesting that maximum biosecurity effectiveness is only achieved through a broader perspective on the subject. In this context, biosecurity includes efforts to minimize the number of fish suppliers and hence the possibilities for introduction of infection into a farm. On the other hand, for freshwater farms the effect of indegree and the interaction between this and biosecurity is not supported by the available data (Table 5).

Movement of live fish has been identified as one of the main routes of introduction of harmful pathogens onto a farm [7, 8], and associated with increased risk for specific diseases such as ISA [41, 42] and IPN [43, 44]. The effect of indegree in reducing the effectiveness of biosecurity could be related to increased instances of fish stress (one for each stocking, potentially many for each supplier), which in turn would make fish more susceptible to disease, and to the multiple chances of introducing infected fish populations to the farm. It seems logical that even the highest biosecurity would become compromised if not coupled with measures to mitigate the risk arising from this source, either through minimization of the number of suppliers and/or through increased biosecurity standards for live fish suppliers including screening and testing of all new fish groups prior to stocking.

Of salmonid farms in Ireland, results would indicate that seawater salmon farms would have the highest biosecurity levels, with freshwater salmon and trout farms having the lowest values (Table 2). However, comparisons between different production settings could be misleading, as the instruments of measurement (surveys) were different for each type of farm, being this in turn a reflection of the different nature of the environments in which these production phases take place. Because of this, results should not be interpreted as indication of increased disease risk in freshwater compared to seawater farms but rather of the potential for improvements in biosecurity in these premises. Therefore, the biosecurity score developed here should be considered a tool of benchmarking farms within the same environment, rather than comparing biosecurity between different production settings.

Environmental exposure via water is probably a more important pathway for pathogen introduction for seawater than freshwater salmon farms [8], especially because most seawater farms (78%) reported sharing a bay with others (supporting information S1 File). Transmission through short seaway distances between farming sites has been supported in studies for pathogens such as Infectious salmon anemia virus (ISAV) [45, 46] and PD [47–49]. On the other hand, most of the freshwater salmon farms (75%) reported not sharing the water supply with other farms (supporting information S2). This could explain the lower pathogen richness found in freshwater salmon farms. The higher pathogen richness in freshwater trout farms (which had a similar score to freshwater salmon farms) could be related to the seemingly less controlled environmental conditions of this type of farms in Ireland. Several of these farms having earth pond rearing systems, with very little, if any, possibility to do a thorough cleaning between generations of fish, a constant mixing of fish generations (only one farm reported to do all-in-all-out production of fish), less control over water quality parameters, and less efficient mortality removal and predator control practices. In addition, 50% of these farms shared water supply with other farms (supporting information S3).

Another possible explanation for the higher pathogen richness in this type of farms is the introduction of these pathogens via contaminated fertilized egg imports, as most of the freshwater trout farms in Ireland import their eggs from abroad, with little movement between farms (Table 2). This hypothesis was not considered in our analyses, as none of the reported pathogens is exotic, and most suppliers and farmers would do a thorough disinfection of the eggs’ surface with iodine disinfectants prior to stocking.

In the case of freshwater salmon farms, most of them received fish from other farms within the country, with only two farms not receiving fish from other farms: one farm imported all of its stock (eggs) from abroad, and the other one was a research facility that participated in a population enhancement program, capturing wild broodstock and releasing juveniles to the environment.

This could explain the lack of evidence of the effect of indegree on pathogen richness for freshwater farms (Table 5, Fig 2), as the freshwater farms with higher variability in indegree (salmon freshwater farms) were the farms with lowest variability in pathogen richness, whereas freshwater trout farms, where variability for pathogen richness was high, had very little variability in terms of indegree (Table 2).

Regarding the specific diseases that comprised the pathogen richness of a seawater farm (Table 3), the most common was pancreas disease. This is a viral infectious disease caused by the salmonid alphavirus pancreas disease virus (PDV) [50, 51]. The second most common disease was amoebic gill disease (AGD) whose causative agent, *Neoparamoeba perurans*, is considered an environmental free-living protozoan [52, 53]. Finally, only one farm reported an outbreak of infectious pancreatic necrosis, which is caused by the IPN virus [54].

The outcomes used in the models presented here (pathogen richness and the specific diseases that compose it) are based on the causative agents having a manifest effect on the fish populations, mortality being the most notable. It is possible that farms that did not report outbreaks of a specific disease were in fact subclinically infected. This might particularly be the case for the diseases of Atlantic salmon seawater sites PD, IPN, and AGD, whose causative agents are widespread in the Irish marine farms [44, 50, 52, 53, 55–57]. The effect of the biosecurity score in the incidence of disease outbreaks caused by these agents could, at least in part, be a reflection of the association between biosecurity and good husbandry practices, which, together with other environmental and host factors, determine whether or not a pathogen causes an overt disease [58]. Similarly, the effect of a seawater salmon farm’s indegree on these diseases could be considered an indicator of fish stress (for both new and resident fish) associated with the stocking of fish, which in turn would affect the ability of fish to resist disease [59]. This is particularly true for AGD’s causative agent, *N*. *perurans*, as this is an environmental free-living protozoan.

For seawater farms, results from the binary logistic regression models (supporting information S4) indicate that biosecurity is not associated with outbreaks of all the evaluated diseases. Only PD showed evidence of association at the 50% PI level. The absence of evidence for the effect of biosecurity on AGD outbreaks would suggest that the occurrence of outbreaks of this disease is not affected by biosecurity practices at the farm level. This was also the case for indegree. This is perhaps because this pathogen is mostly and environmental free-living protozoan [52]. Although indegree and the interaction term did not seem to be associated at 50% PI level in the PD and AGD models, in both models the regression coefficients for these covariates showed mean posteriors above the null value. The mostly positive 50% PI of the effect of AGD on PD, and the virtually null effect of PD on AGD, would indicate that AGD would predispose to the occurrence of PD, but the reverse effect would not happen. This association should be interpreted with caution though, as temporality of the events is not included in the analyzed data.

Regarding the binary logistic regression models of diseases of freshwater that were modeled individually, for BGD the effect of biosecurity would be protective, with no effect of indegree or the interaction term. The apparent protective effect of RTFS on this disease (median: -0.34; 50% PI: -0.66, -0.03) would indicate an antagonistic effect between the respective causative agents: *F*. *psychrophilum* and *F*. *branchiophilum*. This observed antagonistic effect between diseases caused by *Flavobacterium* sp. might be related to similar pathogenesis and susceptibility of these agents to control measures. Presumably, after controlling the outbreak of one of these diseases, the other will be prevented for a certain period of time. This would be supported by the fact that the effect of BGD outbreaks on RTFS is similar (median: -0.27; 50% PI: -0.60, 0.05, supporting information S4).

For the models of *Ichthyobodo* sp. and RTFS, the occurrence of one disease was associated with the occurrence of the other, indicating that these diseases would predispose to each other, or that measures to control one disease do not control the other. This synergistic effect between *Ichthyobodo* sp. and *F*. *psychrophilum* outbreaks could be related to the different mechanisms of pathogenicity between these two infectious agents, with one being a gram-negative bacteria and the other a protozoa, the former causing a septicemic disease and the latter targeting the branchiae and fish surface. One of these pathogens would weaken the host, predisposing to colonization by the other. Mixed infections of *F*. *psychrophilum* and certain viral, bacterial or parasitic fish pathogens are frequently observed in various salmonid fish species [60]. Similarly, it has been shown that a lesser infestation with *Ichthyobodo* sp. can reduce performance and predispose fish to other infectious agents, especially in young fish being reared at high densities. The skin and gill damage resulting from the parasite can be a portal for opportunistic bacteria, fungi, or other pathogens [61].

Regarding the modeling approach used, a generalized Poisson distribution was chosen to capture the lack of independence between different disease events in a farm, due to interaction between different diseases (as discussed above), and in-farm learning curve/change in disease awareness. It also enabled us to flexibly model a count process, allowing for counts that are either under-dispersed (i.e. pathogen richness in seawater farms) or over-dispersed (i.e. pathogen richness in freshwater farms) [22]. Although not perfect, as shown by the slight overestimation in the amount of zero counts and a small proportion of simulated counts greater than observed ones (Fig 5), this model far outperforms a Poisson regression model, indicating that this would be a more suitable model for the data originating process at hand.

Results from the Bayesian generalized Poisson models were in line with results from fitting Poisson regression model with a flexible dispersion parameter ω through quasi-likelihood estimation. Specifically, the SW model showed parameter estimates (p-values) of -0.35 (0.143) for the biosecurity score, 0.56 (0.048) for indegree, and 1.56 (0.005) for the interaction term, with ω = 0.22 indicating under-dispersion. For the FW model, the parameter estimates from the quasi-likelihood model were also similar to the Bayesian model, with parameter estimates (p-values) of -0.50 (0.479) for the biosecurity score, -0.111 (0.886) for indegree, and -0.02 (0.992) for the interaction term, with ω = 1.31 indicating over-dispersion. As expected, the results for the Bayesian models are closer to the null value due to the strong regularizing priors (N(0, 0.5) used to prevent overfitting) [62].

Possible limitations of this study include its cross-sectional nature, where the survey was administered during a two month span, incorporating questions regarding both exposure (biosecurity) and outcome (disease outbreaks) in the year prior to the survey. This would mean that associations found here may not be causal. Nevertheless, biosecurity policies are expected to be relatively constant, with major changes expected only under major disease events, such as the introduction of exotic infectious agents with high associated mortalities.

There are two potential sources of measurement error identified in this study: recall bias and reporting bias [63]. Recall bias could have arisen if managers of farms with disease problems were more likely to identify biosecurity failings, and so reported them more frequently than managers of farms with good disease records. Reporting bias could have occurred considering that support of good biosecurity practices is expected from both public and private entities, such as the Irish Department of Agriculture, Food and the Marine, quality assurance schemes, and farmers’ associations. We believe the latter potential bias was greatly mitigated by the fact that the survey was applied in person at the farm, being preceded by a walk-through and visual inspection of the farm premises by the interviewer.

Additionally, since the recollection of disease events is related to the ascribed cause of mortality, and not necessarily to confirmatory testing for the infectious agent (although this would be the case in most situations), it is possible that misclassification could have sometimes occurred. It is also possible that differences exist between farms in their likelihood of detecting each disease based on their monitoring techniques. This is somewhat reflected in the biosecurity score, as it would be expected that farms with higher score are more thorough in their routines for early disease identification. If anything, this difference in likelihood of detection would bias the associations found here towards the null effect (i.e. as higher biosecurity would be associated with higher pathogen richness). This would mean that associations found here are potentially underestimated. It would also explain the lack of a clear association between biosecurity score and pathogen richness in freshwater farms.

Severity of disease outbreaks is also very variable, with reports for most infectious agents exhibiting a wide range of mortalities [53, 54, 64]. Hence pathogen richness may be only moderately related to severity of disease losses.

Access to farm production records in the country should be considered in future research, including attributed mortality causes, to get records of fish health outcomes with minimum bias. This would further elucidate whether pathogen richness is causally associated with biosecurity and with indegree, and to quantify the association between pathogen richness and fish losses.

Another issue relates to the weight assigned to each question in the survey for estimating the biosecurity score. In the current study, all questions had the same weight. Nonetheless, it is possible that different weighting schemes would produce different results, increasing or decreasing the measure of effect. An attempt was made to identify the most important questions of the survey through matrix factorization, specifically principal component analysis, for the score of seawater salmon farms. Using this approach, the burden of questions was reduced from 108 to 55, and when fitted in a model equivalent to the one using the full data set, we were able to establish a similar interplay between biosecurity score, indegree, and pathogen richness, although the magnitude of the effect estimated with the score based on the full survey was much higher for both the score and the interaction with indegree (results not shown).

The work presented here could be used to elaborate indicators of a farm’s risk of disease based on its biosecurity score and indegree, to inform risk-based disease surveillance and control activities at both the private and public sectors. A similar approach is currently practiced by the Scottish regulator, where a risk-based surveillance scheme is used to define the frequency of veterinary inspections, based on risk factors like the ones included in the survey from which the score was calculated [65] and in the number of fish providers.

## Supporting information

S1 File Marine farms biosecurity survey results. (DOCX)

S2 File Freshwater farms biosecurity survey results. (DOCX)

S3 File FW trout farms biosecurity survey results. (DOCX)

S4 File Binary logistic regression models’ results. (DOCX)

## References

[1] Anon. Domestic live fish movement dataset: 2014. Marine Institute; 2015.

[2] Green DM, Gregory A, Munro LA. Small- and large-scale network structure of live fish movements in Scotland. Prev Vet Med. 2009;91(2–4):261–9. doi: 10.1016/j.prevetmed.2009.05.031 19625093 10.1016/j.prevetmed.2009.05.031

[3] Green DM, Werkman M, Munro LA, Kao RR, Kiss IZ, Danon L. Tools to study trends in community structure: application to fish and livestock trading networks. Prev Vet Med. 2011;99(2–4):225–8. doi: 10.1016/j.prevetmed.2011.01.008 21353716 10.1016/j.prevetmed.2011.01.008

[4] Green DM, Werkman M, Munro LA. The potential for targeted surveillance of live fish movements in Scotland. J Fish Dis. 2012;35(1):29–37. doi: 10.1111/j.1365-2761.2011.01321.x 22168453 10.1111/j.1365-2761.2011.01321.x

[5] Munro LA, Gregory A. Application of network analysis to farmed salmonid movement data from Scotland. J Fish Dis. 2009;32(7):641–4. doi: 10.1111/j.1365-2761.2009.01076.x 19538253 10.1111/j.1365-2761.2009.01076.x

[6] Yatabe T, More SJ, Geoghegan F, McManus C, Hill AE, Martínez-López B. Characterization of the live salmonid movement network in Ireland: Implications for disease prevention and control. Preventive Veterinary Medicine. 2015;122(1–2):195–204. doi: 10.1016/j.prevetmed.2015.09.005 26388525 10.1016/j.prevetmed.2015.09.005

[7] Oidtmann BC, Crane CN, Thrush MA, Hill BJ, Peeler EJ. Ranking freshwater fish farms for the risk of pathogen introduction and spread. Prev Vet Med. 2011;102(4):329–40. doi: 10.1016/j.prevetmed.2011.07.016 21872950 10.1016/j.prevetmed.2011.07.016

[8] Oidtmann BC, Peeler E, Lyngstad T, Brun E, Jensen BB, Stärk KD. Risk-based methods for fish and terrestrial animal disease surveillance. Preventive veterinary medicine. 2013;112(1):13–26. 23948144 10.1016/j.prevetmed.2013.07.008

[9] BIM. Annual Aquaculture Survey 2015 In: Department of Agriculture Fat M, editor. Dublin, Ireland: Bord Iascaigh Mhara, Irish Sea Fisheries Board; 2016 p. 16.

[10] Pruder GD. Biosecurity: application in aquaculture. Aquacultural Engineering. 2004;32(1):3–10.

[11] Oidtmann BC, Thrush MA, Denham KL, Peeler EJ. International and national biosecurity strategies in aquatic animal health. Aquaculture. 2011;320(1–2):22–33.

[12] Mitchell CE, Blumenthal D, Jarošík V, Puckett EE, Pyšek P. Controls on pathogen species richness in plants’ introduced and native ranges: roles of residence time, range size and host traits. Ecology Letters. 2010;13(12):1525–35. doi: 10.1111/j.1461-0248.2010.01543.x 20973907 10.1111/j.1461-0248.2010.01543.x PMC3003901

[13] Arneberg P. Host population density and body mass as determinants of species richness in parasite communities: comparative analyses of directly transmitted nematodes of mammals. Ecography. 2002;25(1):88–94.

[14] Jones KE, Patel NG, Levy MA, Storeygard A, Balk D, Gittleman JL, et al Global trends in emerging infectious diseases. Nature. 2008;451(7181):990–3. doi: 10.1038/nature06536 18288193 10.1038/nature06536 PMC5960580

[15] Dunn RR, Davies TJ, Harris NC, Gavin MC. Global drivers of human pathogen richness and prevalence. Proceedings Biological sciences. 2010;277(1694):2587–95. doi: 10.1098/rspb.2010.0340 20392728 10.1098/rspb.2010.0340 PMC2982038

[16] A code of good practice for Scottish finfish aquaculture, (2006).

[17] Yanong RP. Biosecurity in aquaculture, part 2. Recirculating aquaculture systems Southern Regional Aquaculture Center, Publication. 2012;4708.

[18] Yanong RP. Biosecurity in aquaculture, Part 3: Ponds. SRAC Publication-Southern Regional Aquaculture Center. 2013(4712).

[19] Yanong RP, Erlacher-Reid C. Biosecurity in aquaculture, part 1: an overview. USDA Southern Regional Aquaculture Center. 2012;4707.

[20] Consul PC, Jain GC. A Generalization of the Poisson Distribution. Technometrics. 1973;15(4):791–9.

[21] Scollnik DP. On the analysis of the truncated generalized Poisson distribution using a Bayesian method. Astin Bulletin. 1998;28(01):135–52.

[22] Consul P, Famoye F. Generalized Poisson regression model. Communications in Statistics-Theory and Methods. 1992;21(1):89–109.

[23] Tuenter HJH. On the generalized Poisson distribution. Statistica Neerlandica. 2000;54(3):374–6.

[24] Consul PC. Generalized Poisson distributions: properties and applications. New York: Marcel Dekker Inc; 1989.

[25] Gelman A. Scaling regression inputs by dividing by two standard deviations. Statistics in medicine. 2008;27(15):2865–73. doi: 10.1002/sim.3107 17960576 10.1002/sim.3107

[26] Vehtari A, Gelman A, Gabry J. Practical Bayesian model evaluation using leave-one-out cross-validation and WAIC. Statistics and Computing. 2016:1–20.

[27] Gelman A, Carlin JB, Stern HS, Dunson DB, Vehtari A, Rubin DB. Bayesian data analysis. 3 ed: Chapman & Hall/CRC Boca Raton, FL, USA; 2014.

[28] Gelman A, Rubin DB. Inference from iterative simulation using multiple sequences. Statistical science. 1992:457–72.

[29] Stan Development Team. Stan Modeling Language Users Guide and Reference Manual. Version 2.14.0 ed2016.

[30] R Development Core Team. R: A language and environment for statistical computing. In: Computing , RFfS, editor. Vienna, Austria 2017.

[31] Stan Development Team. RStan: the R interface to Stan. 2.14.1 ed2016.

[32] Vehtari A, Gelman A, Gabry J. loo: Efficient leave-one-out cross-validation and WAIC for Bayesian models. 2016. p. R package version 1.0.

[33] Kleiber C, Zeileis A. Visualizing Count Data Regressions Using Rootograms. The American Statistician. 2016;70(3):296–303.

[34] Kirkpatrick R. RMKdiscrete (Version 0.1). Software and documentation available at http://cranr-projectorg/web/packages/RMKdiscrete. 2014.

[35] Gabry J. bayesplot: Plotting for {Bayesian} models. 2017.

[36] Delabbio J, Murphy B, Johnson GR, Hallerman E. Characteristics of the recirculation sector of finfish aquaculture in the United States and Canada. International Journal of Recirculating Aquaculture. 2003;4:5.

[37] Delabbio J, Murphy BR, Johnson GR, McMullin SL. An assessment of biosecurity utilization in the recirculation sector of finfish aquaculture in the United States and Canada. Aquaculture. 2004;242(1–4):165–79.

[38] Delabbio JL, Johnson GR, Murphy BR, Hallerman E, Woart A, McMullin SL. Fish Disease and Biosecurity: Attitudes, Beliefs, and Perceptions of Managers and Owners of Commercial Finfish Recirculating Facilities in the United States and Canada. Journal of Aquatic Animal Health. 2005;17(2):153–9.

[39] Gunn G, Heffernan C, Hall M, McLeod A, Hovi M. Measuring and comparing constraints to improved biosecurity amongst GB farmers, veterinarians and the auxiliary industries. Preventive veterinary medicine. 2008;84(3):310–23. 18282623 10.1016/j.prevetmed.2007.12.003

[40] Delgado AH, Norby B, Dean WR, McIntosh WA, Scott HM. Utilizing qualitative methods in survey design: Examining Texas cattle producers’ intent to participate in foot-and-mouth disease detection and control. Preventive Veterinary Medicine. 2012;103(2):120–35. 21968089 10.1016/j.prevetmed.2011.09.012

[41] Murray AG, Smith RJ, Stagg RM. Shipping and the spread of infectious salmon anemia in Scottish aquaculture. Emerg Infect Dis. 2002;8(1):1–5. 11749740 10.3201/eid0801.010144 PMC2730283

[42] Mardones FO, Martinez-Lopez B, Valdes-Donoso P, Carpenter TE, Perez AM. The role of fish movements and the spread of infectious salmon anemia virus (ISAV) in Chile, 2007–2009. Prev Vet Med. 2014;114(1):37–46. doi: 10.1016/j.prevetmed.2014.01.012 24485704 10.1016/j.prevetmed.2014.01.012

[43] Jarp J, Gjevre AG, Olsen AB, Bruheim T. Risk factors for furunculosis, infectious pancreatic necrosis and mortality in post-smolt of Atlantic salmon, Salmo solar L. J Fish Dis. 1995;18(1):67–78.

[44] Ruane NM, Murray AG, Geoghegan F, Raynard RS. Modelling the initiation and spread of Infectious Pancreatic Necrosis Virus (IPNV) in the Irish salmon farming industry: The role of inputs. Ecol Model. 2009;220(9–10):1369–74.

[45] Aldrin M, Lyngstad TM, Kristoffersen AB, Storvik B, Borgan Ø, Jansen PA. Modelling the spread of infectious salmon anaemia among salmon farms based on seaway distances between farms and genetic relationships between infectious salmon anaemia virus isolates2011 2011-09-07 00:00:00. 1346–56 p. 10.1098/rsif.2010.0737 PMC3140724 21325314

[46] McClure C, Hammell K, Dohoo I. Risk factors for outbreaks of infectious salmon anemia in farmed Atlantic salmon. Preventive Veterinary Medicine. 2005;72(3–4):263–80. doi: 10.1016/j.prevetmed.2005.07.010 16188335 10.1016/j.prevetmed.2005.07.010

[47] Viljugrein H, Staalstrøm A, Molvær J, Urke HA, Jansen PA. Integration of hydrodynamics into a statistical model on the spread of pancreas disease (PD) in salmon farming. Diseases of Aquatic Organisms. 2009;88:35–44. doi: 10.3354/dao02151 20183963 10.3354/dao02151

[48] Kristoffersen AB, Viljugrein H, Kongtorp RT, Brun E, Jansen PA. Risk factors for pancreas disease (PD) outbreaks in farmed Atlantic salmon and rainbow trout in Norway during 2003–2007. Preventive Veterinary Medicine. 2009;90(1–2):127–36. doi: 10.1016/j.prevetmed.2009.04.003 19419787 10.1016/j.prevetmed.2009.04.003

[49] Tavornpanich S, Paul M, Viljugrein H, Abrial D, Jimenez D, Brun E. Risk map and spatial determinants of pancreas disease in the marine phase of Norwegian Atlantic salmon farming sites. BMC Veterinary Research. 2012;8(1):172. 23006469 10.1186/1746-6148-8-172 PMC3514396

[50] Rodger H, Mitchell S. Epidemiological observations of pancreas disease of farmed Atlantic salmon, Salmo salar L., in Ireland. J Fish Dis. 2007;30(3):157–67. doi: 10.1111/j.1365-2761.2007.00799.x 17352791 10.1111/j.1365-2761.2007.00799.x

[51] Graham DA, Fringuelli E, Wilson C, Rowley HM, Brown A, Rodger H, et al Prospective longitudinal studies of salmonid alphavirus infections on two Atlantic salmon farms in Ireland; evidence for viral persistence. Journal of Fish Diseases. 2010;33(2):123–35. doi: 10.1111/j.1365-2761.2009.01096.x 19732268 10.1111/j.1365-2761.2009.01096.x

[52] Downes JK, Henshilwood K, Collins EM, Ryan A, O’Connor I, Rodger HD, et al A longitudinal study of amoebic gill disease on a marine Atlantic salmon farm utilising a real-time PCR assay for the detection of Neoparamoeba perurans. Aquaculture Environment Interactions. 2015;7(3):239–51.

[53] Mitchell SO, Rodger HD. A review of infectious gill disease in marine salmonid fish. Journal of Fish Diseases. 2011;34(6):411–32. doi: 10.1111/j.1365-2761.2011.01251.x 21401646 10.1111/j.1365-2761.2011.01251.x

[54] Roberts RJ, Pearson MD. Infectious pancreatic necrosis in Atlantic salmon, Salmo salar L. J Fish Dis. 2005;28(7):383–90. doi: 10.1111/j.1365-2761.2005.00642.x 16083443 10.1111/j.1365-2761.2005.00642.x

[55] Ruane N, Geoghegan F, Ó Cinneide M. Infectious Pancreatic Necrosis Virus and Its Impact on the Irish Salmon Aquaculture and Wild Fish Sectors. Galway: Marine Institute; 2007.

[56] Ruane NM, McCarthy LJ, Swords D, Henshilwood K. Molecular differentiation of infectious pancreatic necrosis virus isolates from farmed and wild salmonids in Ireland. J Fish Dis. 2009;32(12):979–87. doi: 10.1111/j.1365-2761.2009.01080.x 19602095 10.1111/j.1365-2761.2009.01080.x

[57] Ruane N, Rodger H, Mitchell S, Doyle T, Baxter E, Fringuelli E. GILPAT—An Investigation into Gill Pathologies in Marine Reared Finfish. Galway, Ireland 2013.

[58] Plumb JA, Hanson LA. Health maintenance and principal microbial diseases of cultured fishes: John Wiley & Sons; 2011.

[59] Bernoth E-M, Ellis AE, Midtlyng PJ, Olivier G, Smith P. Furunculosis: multidisciplinary fish disease research: Academic Press; 1997.

[60] Cipriano RC, Holt RA. Flavobacterium psychrophilum, cause of bacterial cold-water disease and rainbow trout fry syndrome: US Department of the Interior, US Geological Survey, National Fish Health Research Laboratory; 2005.

[61] Farmer BD, Straus DL, Mitchell AJ, Beck BH, Fuller SA, Barnett LM. Comparative Effects of Copper Sulfate or Potassium Permanganate on Channel Catfish Concurrently Infected with Flavobacterium columnare and Ichthyobodo necator. Journal of Applied Aquaculture. 2014;26(1):71–83.

[62] McElreath R. Statistical rethinking: A Bayesian course with examples in R and Stan: CRC Press; 2016.

[63] Gordis L. Epidemiology. 4th ed: Saunders; 2009 400 p.

[64] Kilburn R, Murray A, Hall M, Bruno D, Cockerill D, Raynard R. Analysis of a company′s production data to describe the epidemiology and persistence of pancreas disease in Atlantic salmon (Salmo salar L.) farms off Western Scotland. Aquaculture. 2012;368:89–94.

[65] Anon. Surveillance Programme Fish Health Inspectorate: Marine Scotland Directorate; 2017 [updated 01/21/2016; cited 2017 08/22/2017]. http://www.gov.scot/Topics/marine/Fish-Shellfish/FHI/surveillance.

