# Supplementary material for: Can biosecurity and local network properties predict pathogen species richness in the salmonid industry?
Source: PLoS One. 2018 Jan 30;13(1):e0191680. doi: 10.1371/journal.pone.0191680 (PMC5790274; doi:10.1371/journal.pone.0191680)
Supplement: S1 File — (DOCX) [file pone.0191680.s001.docx]

**SEAWATER SALMON FARMS BIOSECURITY SURVEY RESULTS (18 FARMS)**

1. ***SITE STOCKING***

**15) What is the initial typical stocking density in the site?**

**15.1) What is the final typical stocking density in the site?**

Stocking density was not reported uniformly through the farms. In order to estimate it would require far too many assumptions. Hence this question was not further considered.

**B) *PREDATOR CONTROL***

**3) Does your site have an SOP for prevention and management of predation problems?**

100% of sites have SOPs for prevention and management of predation problems

1. ***CLEANER FISH***

**6) Before re-utilizing cleaner fish, do you run diagnostic tests on them for diseases of concern for salmon?**

All of the sites (3) that re-cycled the cleaner fish reported to run diagnostic tests for diseases of concern of salmon

1. ***DISEASE PREVENTION AND CONTROL***

**6) Do you have a classification scheme for mortalities?**

100% of sites have classification schemes for mortalities

1. ***DIVERS, DIVING EQUIPMENT AND MORTALITIES HANDLING***
2. **What is the normal frequency of diving at your site?**

100% of sites had diving frequencies of at least 2 times a week

1. **In the event of an outbreak, would the frequency of diving change?**

100% of sites would increase the frequency of diving when an outbreak occurs

1. **How frequently would diving take place in the event of an outbreak?**

100% of sites would have daily diving in the case of an outbreak

**6) How are cleaning and disinfection procedures for divers and their equipment enforced?**

100% of sites do cleaning and disinfection procedures for divers and their equipment on site

1. ***FEED AND SITE MANAGEMENT***

**7) Does the site share equipment with other sites (same or different company)?**

100% of sites shared equipment with other sites.

**14) What are the disinfectants of choice in your facility?**

The most used disinfectant were Actiplus™, Virkon™, Iosan™, Virafor™, and Virasure™. These were used for nets, pens, cages, farm equipment, surfaces, floors, transports, and footbaths. For hands, most farms used hand sanitizer.

1. ***HARVESTING***

**2.2) During harvesting is blood and water effectively disinfected?**

100% of managers consider that during harvesting blood and water were effectively disinfected.

**6) Is the site fallowed after harvesting as a regular practice (i.e. > 75% of the time)?**

100% of sites reported to be fallowed after harvesting as a regular practice

**7. 1) Exactly (weeks)?**

The median fallowing time was 8 weeks, while the first and third quartile were 5.5 and 12 weeks, respectively.

1. ***VACCINES***

Some sites reported not to know what vaccines, so I need to see vaccination regime of FW sites supplying to these sites.

**Questions 3 through 7**: Sites did not vaccinate during a production cycle, so these questions don’t apply.

1. **COORDINATED BAY MANAGEMENT**

Since many sites that shared the bay with sites from same company, they declared that the above aspects were company policies shared by all of the sites.

Other(s): these sites coordinated sea lice treatments

**5) With what frequency do you evaluate the efficacy of sea lice monitoring and treatment at your site (or within the bay your site is located in, if coordinated with other sites)?**

100% of managers said they evaluate the efficacy of sea lice monitoring and treatment in their sites/bays at least yearly. When asked about the detail they responded this:

It is possible that the question was misunderstood here, and what they answer was how frequently they assessed the effect of treatments against sea lice (instead of evaluating the of efficacy the strategy of sea lice monitoring and treatment, which was the original intent). Still useful though, as the answer provided most likely reflects the frequency of sea lice counts.

18% of all sites reported to treat every 2 weeks.

Some sites reported both hydrogen peroxyde and freshwater as treatments of choice. This seems to be related to AGD control.

1. ***FISH WELFARE AND CARE***
   1. **Regarding the site you manage: does it have an adequate supply of water of suitable quality at all times?**

100% of site managers strongly agreed on that their sites had an adequate supply of water of suitable quality at all times

**2) With which frequency is water quality checked at your site?**

100% of sites check water quality (e.g. turbidity, temperature, salinity, oxygen levels) on a daily basis, while plankton was checked either weekly or 2 times a week for most sites (one site will only do it if they see something wrong).

**6) Do you have written feed withdrawal protocols?**

100% of sites had written feed withdrawal protocols (although for some this was a new practice).

*Every 2 weeks refers to net cleaning.

Some sites did not specify season, so the same frequency was assigned to both summer and winter. This wuold explain every 2 weeks frequency during winter.

**K) *MANAGEMENT OF PEOPLE***

**1) Are there disinfection stations for people?**

100% of sites had disinfections stations for people.

1. ***BIOSECURITY PROGRAM AND RECORDS***

**1) Do you have standard operating procedures (SOPs) for biosecurity?**

100% of sites have standard operating procedures (SOPs) for biosecurity

**2) Are personnel trained on the importance of biosecurity, SOPs (if available), and compliance on a regular basis?**

100% of the farms declared personnel are trained on a regular basis.

1. **How often do you review your biosecurity program, SOPs (if available) and employee compliance?**

100% of farms review their biosecurity program at least on a yearly basis.

1. **Is there a biosecurity manager in your facility (i.e. who oversees the biosecurity program and helps to develop it)?**

94% of sites have a biosecurity manager (usually is the farm manager).

1. **How often are biosecurity measures on your site audited?**

100% of sites are audited on their biosecurity measures at least yearly.
