# Supplementary material for: Can biosecurity and local network properties predict pathogen species richness in the salmonid industry?
Source: PLoS One. 2018 Jan 30;13(1):e0191680. doi: 10.1371/journal.pone.0191680 (PMC5790274; doi:10.1371/journal.pone.0191680)
Supplement: S2 File — (DOCX) [file pone.0191680.s002.docx]

**FRESHWATER SALMON FARMS BIOSECURITY SURVEY RESULTS (8 FARMS)**

1. ***SITE STOCKING***

**2) What is the main source of water for your facility?**

All sites used surface water (lake, river, stream) as their primary source of water

**3) Are there other water source(s)?**

25% (2) sites had a secondary water source: 1 used underground water from eggs to first feeding, and another one used pumped seawater (broodstock)

**4) Are there any other fish farms or fish processors that share the same water supply?**

25% of sites had another fish farm sharing the same water supply

**6) Can individual rearing units be taken off-line and isolated in the event of a disease outbreak?**

88% (7) of sites could take off-line and isolate individuals units in the case of a disease outbreak

**7) Do you have available on your site back-up components for your rearing units?**

75% (6) of sites have back-up components for the rearing units

**8) In your facility, are different sections or areas separated by physical barriers?**

63% (5) of sites ha physical barriers separating different sections of the farm

**9) What are the typical stocking densities in your facility?**

Stocking density was not reported uniformly through the farms. In order to estimate it would require far too many assumptions. Hence this question was not further considered.

**12) When you receive new fish or eggs from an outside source, do you place them in a quarantine facility / area?**

25% of farms place new fish in a quarantine are/facility. Although several farms receive fish only during a short period of time during the production cycle

**13) How long does the quarantine period last in your facility?**

The two sites that have quarantine, do it for 8 and 12 weeks, respectively

**15) Do you have written protocols for movement of new fish onto the facility and between areas of the facility?**

75%(6) of sites had protocols for movement of fish

**16) What type of transport do you use for sending/receiving shipments of fish?**

38% (3) of sites use only third company transport, 63% (5) use both own company and third company transports

**17) For third party transporters, do you check the transporter is registered with the Marine Institute?**

All sites checked that third party transport were registered with the MI

1. ***DISEASE PREVENTION AND CONTROL***
2. **All farms check their fish at least once a day**

**3.2) With what frequency are mortalities picked out at your facility under normal circumstances?: Fry-parr**

All farms do it at least daily

**5) Are the fish stocked in this site vaccinated at the site of origin?**

Of the farms that received fish in stages other than eggs (4 farms), 25% (1) received vaccinated fish

**5.1) If yes, with what vaccine(s)**

These fish were vaccinated with Alphaject 2-2, PD3, and Aquavac

**6) During a typical production cycle, are stocked fish vaccinated on your site?**

50% (4) of sites vaccinated fish during the production cycle

**7.1) What proportion of the fish are vaccinated?: Fry**

0%

**7.2) What proportion of the fish are vaccinated?: Parr**

Of the 4 sites that vaccinated, all did at the parr stage, 3 vaccinated 100% of its parr, and 1 vaccinated 40%

**7.3) What proportion of the fish are vaccinated?: Smolt**

0%

**9) Is this vaccination audited by an external party?**

100% of farms that vaccine their fish use external audits for vaccination

**10) Do you have a contingency plan in place for disease outbreaks and increased mortalities?**

88% of farms have a contingency plan for such events

**12) Have you had any infectious disease on your fish in the last 12 months?**

25% (2) of the farms have

**13) Can you tell us what disease(s)?**

One farm had furunculosis, the other had gill disease, costia, and fungus

1. ***MANAGEMENT OF PEOPLE***
2. **In your facility, do you have disinfection stations for people?**

100% of sites had disinfection stations for people

**6) Is protective clothing specific to sections or areas of your facility?**

12.5% (1) of sites had clothing specific to certain areas

**8) For staff members of your facility, is access restricted to certain areas?**

25% (2) of sites had restricted access for staff members. One site had only one staff member (owner)

**8.1) If yes, which area(s) have restricted access?**

Of the one with restricted access, they both restricted access to the hatchery, one specifically where imported eggs were put

**9) Are personnel assigned exclusively to specific sections or areas based on age of fish and/or disease status?**

25% (2), the ones with restricted access, sites had personnel exclusively dedicated to specific areas

**10) What would be the order in which they routinely handle the fish of the facility?**

Of the farms that answered this question (5), all will leave disease, quarantine (if present), and broodstock (if present) fish last in their routine

**11) Is there additional, clearly marked and dedicated equipment for diseased/quarantined tanks or systems?**

50% (5) farms had dedicated equipment for diseased and/or quarantined fish

**12) Do you share staff with other sites (same or different company)?**

25% (2) farms shared staff with other farms

**13) What specific measures are in place for personnel arriving from a different site?**

Both sites that shared staff used change of footwear, hand sanitizer, and footbaths. Only one did change of overall/apron. None used showers

**17) Are there areas of the facility that are restricted to vehicles?**

87.5% (7) farms had restrictions of access for vehicles

D) ***SITE AND FEED MANAGEMENT***

**1) Does your site share equipment with other sites (same or different company)?**

37.5% (3) of farms shared equipment with other farms

**2) How do you enforce cleaning and disinfection procedures for equipment that enters the site?**

100% (3) of these farms did their our own cleaning and disinfection on site, and 33% (1) of farms checked and signed OK disinfection protocols, before equipment enters the premises

**4) Do you have a cleaning and disinfection protocol for vehicles that enter/exit the premises?**

71.4% (5) have a cleaning and disinfection protocol for vehicles

**6) Do you have a pest control program in place in the facility?**

87.5%(7) of farms have a pest control on place. One uses cats

**8) How often do you clean and disinfect surfaces on your site**

50% (4) farms do it every time these are used, the other 50% do it only when necessary

**9) For the hatchery, how often are the floors cleaned and disinfected?**

25% (1) of the farms do it at least once a week, 62.5% (5) do it only when necessary

**11) Is equipment used in contact with fish or water specific to certain areas of the facility, based on age of fish and/or their disease status?**

75% (6) of the farms had area-specific equipment

**12) Is all equipment used within each zone of your facility cleaned and disinfected within the zone?**

87.5% (7) farms cleaned and disinfected equipment within their zone of use

**16) What are the disinfectants of choice in your facility?**

Peradox™ for rearing units, equipment, surfaces, floors, transports, and footbaths. Hand sanitizer for hands, and Buffodine™ for eggs

**17) Is the fish feed delivered by the feed company?**

25% (2) sites feed is delivered by feed company

1. ***BIOSECURITY PROGRAM AND RECORDS***
2. **Do you have standard operating procedures (SOPs) for biosecurity?**

100% of sites did have a biosecurity SOP

1. **Are personnel trained on the importance of biosecurity, SOPs (if available), and compliance on a regular basis?**

87.5% (7) farms stated their personnel are trained on these issues.

**3) When was the last time they were trained? (years ago)**

The mean was 1.2 years before the interview

**6) Is there a biosecurity manager in your facility (i.e. who oversees the biosecurity program and helps to develop it)?**

75% (6) of the farms have a biosecurity manager (usually was the site manager)
