# Supplementary material for: Can biosecurity and local network properties predict pathogen species richness in the salmonid industry?
Source: PLoS One. 2018 Jan 30;13(1):e0191680. doi: 10.1371/journal.pone.0191680 (PMC5790274; doi:10.1371/journal.pone.0191680)
Supplement: S3 File — (DOCX) [file pone.0191680.s003.docx]

**FRESHWATER TROUT FARMS BIOSECURITY SURVEY RESULTS (8 FARMS)**

1. ***SITE STOCKING***

**2) What is the main source of water for your facility?**

100% (8) of the farms used surface water (lake, river, stream)

**3) Are there other water source(s)?**

No additional water sources were used

**4) Are there any other fish farms or fish processors that share the same water supply?**

50% (4) of farms shared the same water supply with other farms (they shared with each other)

**5) Do you have an influent water treatment?**

There were no farms with influent water treatment system of any kind

**6) Can individual rearing units be taken off-line and isolated in the event of a disease outbreak?**

Only 1 farm (25%) of the farms could isolate their rearing units in the event of disease outbreak

**7) Do you have available on your site back-up components for your rearing units?**

75% (6) of farms have back-up components for the rearing units

**8) In your facility, are different sections or areas separated by physical barriers?**

62.5% (5) of the farms have physical barriers between sections/areas of the farm

**9) What are the typical stocking densities in your facility?**

Stocking density was not reported uniformly through the farms. In order to estimate it would require far too many assumptions. Hence this question was not further considered .

**10) Do you receive fish and/or eggs from a well-known reputable supplier?**

All farms strongly agree on this point

**12) When you receive new fish or eggs from an outside source, do you place them in a quarantine facility / area?**

None of the sites did this, and only one of this practiced an all-in all-out for every generation of fish

**15) Do you have written protocols for movement of new fish onto the facility and between areas of the facility?**

62.5% (5) of the farms have a written protocol for fish movement, of these, 1 only did for moving fish into the farm

**17) For third party transporters, do you check the transporter is registered with the Marine Institute?**

100% of responding farms (5) did check MI registry. 3 did not know

1. ***DISEASE PREVENTION AND CONTROL***
2. **With what frequency are fish in your facility observed for abnormalities**

All farms watch fish daily

2 farms did not receive eggs

**3.2) With what frequency are mortalities picked out at your facility under normal circumstances?: Fry-0+**

100% of farms do it at least daily

**3.3) With what frequency are mortalities picked out at your facility under normal circumstances?: ≥1+**

100% of farms do it at least daily. 2 farms didn’t keep 1+ fish

**4) Which one of the following, best describes the normal method of mortalities disposal for your facility?**

87.5% (7) of farms used a rendering company, 1 farm used a lime pit

**5) Are the fish stocked in this site vaccinated at the site of origin?**

100% of farms that receive fish older than eggs, received vaccinated fish

**5.1) If yes, with what vaccine(s)**

Only two sites knew the vaccines used at origin: they both were for ERM

**6) During a typical production cycle, are stocked fish vaccinated on your site?**

Only 25% (2) of the farms vaccinated fish during the production cycle

**7.1) What proportion of the fish are vaccinated?: Fry**

These two farms vaccinated at this stage

**8) With what vaccine(s)?**

Both vaccinated against ERM

**9) Is this vaccination audited by an external party?**

None audited vaccinations, because they were not parenterally administered

**10) Do you have a contingency plan in place for disease outbreaks and increased mortalities?**

75% (6) of the farms have a contingency plan for disease outbreaks

**12) Have you had any infectious disease on your fish in the last 12 months?**

87.5% (7) of the farms had at least an outbreak in the past 12 months

1. ***MANAGEMENT OF PEOPLE***
2. **In your facility, do you have disinfection stations for people?**

100% of the farms have disinfection stations for people

**6) Is protective clothing specific to sections or areas of your facility?**

50% (3) of the farms that replied (6) to this question have section-specific clothing

**8) For staff members of your facility, is access restricted to certain areas?**

37.5% (3) of the farms had restricted access policies. One farm reported to have only one staffer, which made this policy unfeasible

**8.1) If yes, which area(s) have restricted access?**

Of these 3 farms, 2 did not mixed personnel from the fish processing plant with the farm, the other had restricted access to the hatchery

**9) Are personnel assigned exclusively to specific sections or areas based on age of fish and/or disease status?**

40% (2) of farms that replied to this question (5) do assign personnel this way

**10) What would be the order in which they routinely handle the fish of the facility?**

6 farms would treat diseased fish last in their routine, 2 would do it first thing. For those that held different age groups, they would go from younger to older fish during their routine

**11) Is there additional, clearly marked and dedicated equipment for diseased/quarantined tanks or systems?**

50% (4) of the farms have dedicated equipment for diseased fish

**12) Do you share staff with other sites (same or different company)?**

87.5% (7) of the farms share staff

**17) Are there areas of the facility that are restricted to vehicles?**

75% (6) of the farms had restricted access to vehicles in certain areas

1. ***SITE AND FEED MANAGEMENT***
2. **Does your site share equipment with other sites (same or different company)?**

There were no farms that share equipment

1. **How do you enforce cleaning and disinfection procedures for equipment that enters the site?**

It does not apply, as none share equipment

**4) Do you have a cleaning and disinfection protocol for vehicles that enter/exit the premises?**

100% of the farms have a cleaning and disinfection protocol

**6) Do you have a pest control program in place in the facility?**

100% of the farms have a pest control program in place

2 farms reported not to have surfaces to clean and disinfect, so did not answer this question

**9) For the hatchery, how often are the floors cleaned and disinfected?**

100% (5) of the farms that answered this question clean and disinfect the hatchery floors at least once a week. 3 farms did not answered this question, as they didn’t have a hatchery

**11) Is equipment used in contact with fish or water specific to certain areas of the facility, based on age of fish and/or their disease status?**

75% (6) of the farms had area-specific equipment

**12) Is all equipment used within each zone of your facility cleaned and disinfected within the zone?**

62.5% (5) of the farms cleaned and disinfected the equipment within the zone it was used

**16) What are the disinfectants of choice in your facility?**

Virasure™ was the most frequent disinfectant for rearing units, equipment, surfaces, sloors, and footbaths. For transports the more frequents were Virasure™ and Virkon™, for the hands the most used was hand sanitizer, and for eggs it was Buffodine™

**17) Is the fish feed delivered by the feed company?**

37.5% (3) of the farms have the feed delivered by the feed company

1. ***BIOSECURITY PROGRAM AND RECORDS***
2. **Do you have standard operating procedures (SOPs) for biosecurity?**

All farms have an SOP for biosecurity

1. **Are personnel trained on the importance of biosecurity, SOPs (if available), and compliance on a regular basis?**

75% (6) of the farms train their personnel on biosecurity on a regular basis

1. **When was the last time they were trained? (years ago)**

All of the 6 farms that train their personnel have done it within the last year

**6) Is there a biosecurity manager in your facility (i.e. who oversees the biosecurity program and helps to develop it)?**

100% of the farms have a biosecurity manager, which is usually the farm manager

1. ***Harvesting*** (2 farms only)
2. **Are fish harvested at this facility?**

Only 2 farms harvest fish on site

1. **Are fish processed at this facility?**

Both of these farms process fish within the site

1. **Is the area where fish are processed physically separated from the rest of the site?**

For both farms, the processing takes place in a processing plant within the site, separated from the farm

1. **Does the site process fish from others?**

Both farms receive fish from other farms for processing

1. **How are harvesting residues (e.g. blood, offal, trims) handled?**

Both farms use a rendering company to dispose of the residues
