# Supplementary material for: Can biosecurity and local network properties predict pathogen species richness in the salmonid industry?
Source: PLoS One. 2018 Jan 30;13(1):e0191680. doi: 10.1371/journal.pone.0191680 (PMC5790274; doi:10.1371/journal.pone.0191680)
Supplement: S4 File — (DOCX) [file pone.0191680.s004.docx]

**Binary logistic regression model results**

**Pathogens of seawater farms (N = 17)**

Table 1. Parameter estimates of binary logistic regression models for pancreas disease and AGD outbreaks

| **SW farms: PD** | | | | | | |
| --- | --- | --- | --- | --- | --- | --- |
| **Parameter** | **mean** | **SD** | **2.5%** | **25%** | **75%** | **97.5%** |
| Intercept | 0.63 | 0.39 | -0.13 | 0.37 | 0.89 | 1.40 |
| Biosecurity score | -0.36 | 0.46 | -1.24 | -0.67 | -0.04 | 0.53 |
| Indegree | 0.16 | 0.45 | -0.73 | -0.14 | 0.47 | 1.05 |
| Interaction | 0.18 | 0.48 | -0.75 | -0.13 | 0.51 | 1.13 |
| AGD | 0.26 | 0.43 | -0.59 | -0.04 | 0.55 | 1.09 |
| **SW farms: AGD** | | | | | | |
| Intercept | 0.15 | 0.39 | -0.62 | -0.11 | 0.42 | 0.92 |
| Biosecurity score | 0.21 | 0.46 | -0.69 | -0.10 | 0.51 | 1.10 |
| Indegree | 0.22 | 0.44 | -0.66 | -0.08 | 0.52 | 1.09 |
| Interaction | 0.18 | 0.49 | -0.78 | -0.15 | 0.51 | 1.14 |
| PD | 0.07 | 0.41 | -0.75 | -0.21 | 0.34 | 0.88 |


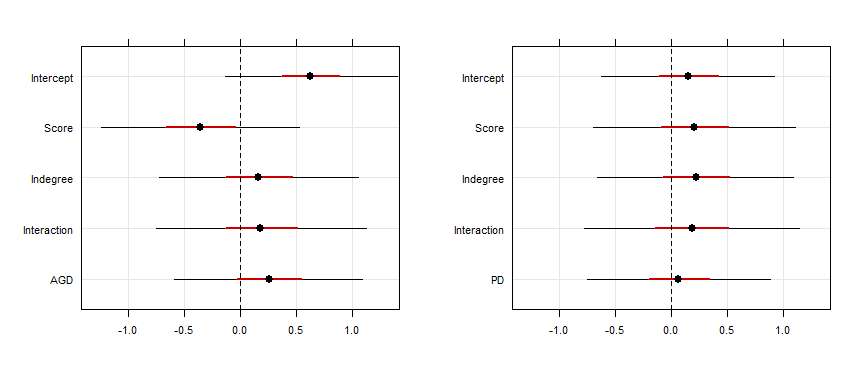


Figure 1. Parameter estimates of binary logistic regression model for PD (left) and AGD (right) outbreaks. Red line 50% PI, black line 95% PI

**Pathogens of freshwater farms (N = 17)**

Table 2. Parameter estimates of binary logistic regression model for BGD, *Ichthyobodo* sp., and RTFS outbreaks

| **FW farms: BGD** | | | | | | |
| --- | --- | --- | --- | --- | --- | --- |
| **Parameter** | **mean** | **SD** | **2.5%** | **25%** | **75%** | **97.5%** |
| Intercept | -0.64 | 0.37 | -1.36 | -0.9 | -0.39 | 0.09 |
| Biosecurity score | -0.35 | 0.45 | -1.24 | -0.66 | -0.05 | 0.54 |
| Indegree | -0.15 | 0.46 | -1.07 | -0.46 | 0.16 | 0.77 |
| Interaction | 0.05 | 0.49 | -0.92 | -0.28 | 0.38 | 1.02 |
| *Ichthyobodo* sp. | -0.11 | 0.46 | -1.03 | -0.42 | 0.21 | 0.79 |
| RTFS | -0.34 | 0.46 | -1.25 | -0.66 | -0.03 | 0.57 |
| **FW farms: *Ichthyobodo* sp.** | | | | | | |
| Intercept | -0.49 | 0.37 | -1.23 | -0.74 | -0.24 | 0.23 |
| Biosecurity score | -0.06 | 0.46 | -0.97 | -0.36 | 0.24 | 0.86 |
| Indegree | -0.03 | 0.46 | -0.92 | -0.34 | 0.28 | 0.88 |
| Interaction | -0.04 | 0.49 | -1.01 | -0.37 | 0.29 | 0.93 |
| BGD | -0.04 | 0.47 | -0.97 | -0.35 | 0.28 | 0.87 |
| RTFS | 0.40 | 0.45 | -0.48 | 0.10 | 0.71 | 1.28 |
| **FW farms: RTFS** | | | | | | |
| Intercept | -0.48 | 0.36 | -1.20 | -0.73 | -0.23 | 0.24 |
| Biosecurity score | 0.03 | 0.45 | -0.84 | -0.27 | 0.33 | 0.91 |
| Indegree | -0.04 | 0.46 | -0.95 | -0.36 | 0.26 | 0.85 |
| Interaction | -0.06 | 0.50 | -1.04 | -0.39 | 0.27 | 0.92 |
| *Ichthyobodo* sp. | 0.41 | 0.45 | -0.46 | 0.11 | 0.71 | 1.29 |
| BGD | -0.27 | 0.48 | -1.21 | -0.60 | 0.05 | 0.66 |


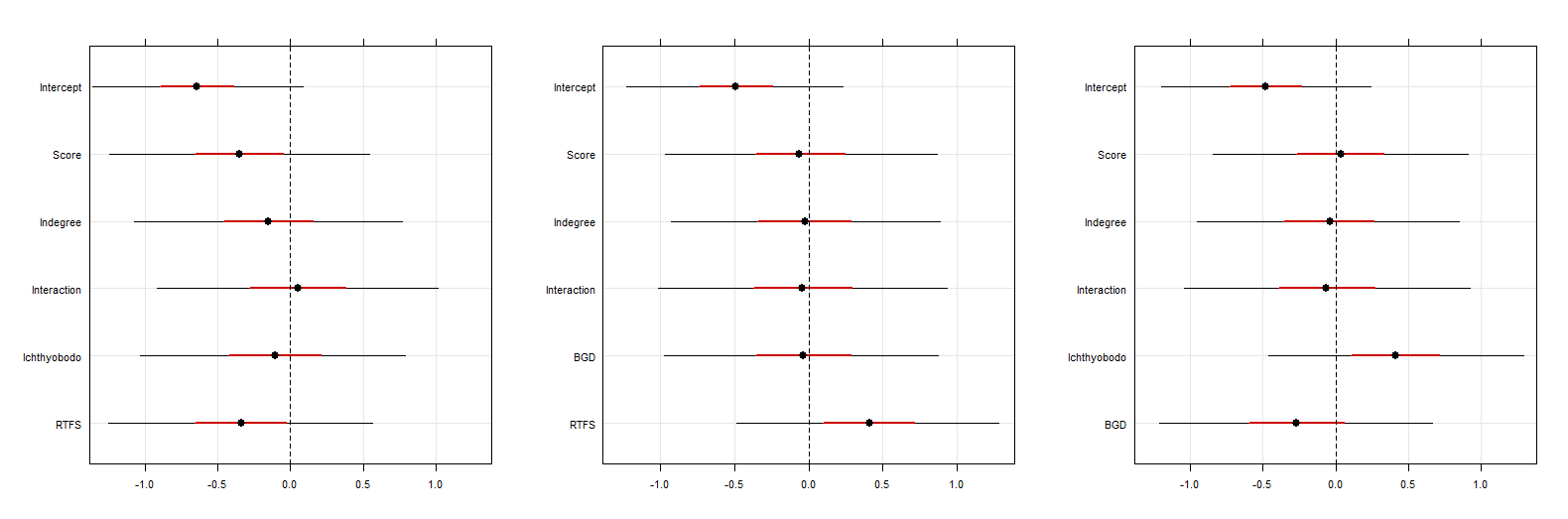
Figure 2. Parameter estimates of binary logistic regression model for bacterial gill disease (left), *Ichthyobodo* sp. (middle), and rainbow trout fry syndrome (right) outbreaks. Red line 50% PI, black line 95% PI
